# Privacy-Preserving Design of Scalar LQG Control

**DOI:** 10.3390/e24070856

**Published:** 2022-06-22

**Authors:** Edoardo Ferrari, Yue Tian, Chenglong Sun, Zuxing Li, Chao Wang

**Affiliations:** 1 School of Electronics and Information Engineering, Tongji University, Shanghai 201804, China; edoardo.ferrari2@studio.unibo.it (E.F.); 2132995@tongji.edu.cn (Y.T.); sunchenglong@tongji.edu.cn (C.S.); chaowang@tongji.edu.cn (C.W.); 2 School of Electrical, Electronic, and Information Engineering “Guglielmo Marconi”—DEI, University of Bologna, 40136 Bologna, Italy

**Keywords:** control–privacy trade-off, hypothesis testing, Kullback–Leibler divergence, optimal control policy, privacy risk analysis

## Abstract

This paper studies the agent identity privacy problem in the scalar linear quadratic Gaussian (LQG) control system. The agent identity is a binary hypothesis: Agent A or Agent B. An eavesdropper is assumed to make a hypothesis testing the agent identity based on the intercepted environment state sequence. The privacy risk is measured by the Kullback–Leibler divergence between the probability distributions of state sequences under two hypotheses. By taking into account both the accumulative control reward and privacy risk, an optimization problem of the policy of Agent B is formulated. This paper shows that the optimal deterministic privacy-preserving LQG policy of Agent B is a linear mapping. A sufficient condition is given to guarantee that the optimal deterministic privacy-preserving policy is time-invariant in the asymptotic regime. It is also shown that adding an independent Gaussian random process noise to the linear mapping of the optimal deterministic privacy-preserving policy cannot improve the performance of Agent B. The numerical experiments justify the theoretic results and illustrate the reward–privacy trade-off.

## 1. Related Work

During the last decades, control technologies have been widely employed and significantly improved the industry productivity, management efficiency, and life convenience. The breakthrough of the deep reinforcement learning (DRL) technology [1] enables the control systems to be intelligent and applicable for more complicated tasks. Along with the increasing concerns about information security and privacy, adversarial problems in control systems have also attracted increasing attentions recently.

The related works and literature are introduced and discussed in the following. There are two types of adversarial problems considered in these works: active attacks and privacy problems.

### 1.1. Research on Active Adversarial Attacks

Most previous works focus on studying the active adversarial attacks on the control systems, which aim to degenerate the control efficiency, or even worse, to lead the system to an undesired state, and developing the corresponding defense mechanisms. Depending on their methodologies, these works can be divided into two classes. One class aims to develop the adversarial reinforcement learning algorithm under attack. The other class makes a theoretic study on the adversarial problem in the standard control model.

DRL takes advantage of the deep network to represent a complex non-linear value function or policy function. Similar to the deep network, DRL is also vulnerable to the adversarial example attack, i.e., the DRL-trained policy can be misled to take a wrong action by adding a minor distortion to the observation of the agent [2]. In [2,3,4,5], the optimal generation of adversarial examples has been studied for given DRL algorithms. As a countermeasure, the mechanism of adversarial training uses adversarial examples in the training phase to enhance the robustness of control policy under attack [6,7,8]. In [9,10], attack/robustness-related regularization terms are added in the optimization objective to improve the robustness of the policy.

In most theoretic studies, adversarial attack problems are modeled from the game theoretic perspective. Stochastic game (SG) [11] and partially observable SG (POSG) can model the indirect (In SG or POSG, players indirectly interact with each other by feeding their actions back to the dynamic environment.) interactions between multiple players in the dynamic control system and have been employed in the robust or adversarial control studies [12,13,14]. Cheap talk game [15] models direct (In the cheap talk game, the sender with private information sends a message to the receiver and the receiver takes an action based on the received message and a belief on the inaccessible private information.) interactions between a sender and a receiver. In [16,17,18,19], the single-step cheap talk game has been extended to dynamic cheap talk games to model the adversarial example attacks in the multi-step control systems. With uncertainty about the environment dynamics in a partially observable Markov decision process (POMDP), the robust POMDP is formulated as a Stackelberg game in [20], where the agent (leader) optimizes the control policy under the worst-case assumption of the environment dynamics (follower). Another kind of adversarial attack maliciously falsifies the agent actions and feeds the falsified actions back to the dynamic environment to degrade the control performance. The falsified action attack can be modeled by Stackelberg games [21,22], where the dynamic environment is the leader and the adversarial agent is the follower. In our previous work [23], the falsified action attack on the linear quadratic regulator control is modeled by a dynamic cheap talk game and the adversarial attack is evaluated by the Fisher information between the random agent action and the falsified action.

Optimal stealthy attacks have also been studied. In [24,25], Kullback–Leibler divergence is used to measure the stealthiness of the attacks on the control signal and the sensing data, respectively; then the optimal attacks against LQG control system are developed with the objective of maximizing the quadratic cost while maintaining a degree of attack stealthiness.

### 1.2. Research on Privacy Problems

Besides the active attacks, passive eavesdropping in control systems leads to privacy problems. Most works focus on preserving the privacy-sensitive environment states. The design of agent actions in the Markov decision process has been investigated when the equivocation of states given system inputs and outputs is imposed as the privacy-preserving objective [26]. In [27,28,29,30], the notion of differential privacy [31] is introduced in the multi-agent control, where each agent adds privacy noise to his states before sharing them with other agents while guaranteeing the whole control system network to operate well. The reward function is a succinct description of the control task and is strongly relevant with the agent actions. The DRL-learned value function can reveal the privacy-sensitive reward function. Regarding this privacy problem, functional noise is added to the value function in the Q-learning such that the neighborhood reward functions are indistinguishable [32]. As a promising computational secrecy technology, labeled homomorphic encryption has been employed to encrypt the private states, gain matrices, control inputs, and intermediary steps in the cloud-outsourced LQG [33].

## 2. Introduction

### 2.1. Motivation

In this paper, we consider the agent identity privacy problem in the LQG control, which is motivated by the inverse reinforcement learning (IRL). IRL algorithms [34] can reconstruct the reward functions of agents and therefore can also be maliciously exploited to identify the agents. Similar to many other privacy problems in the big data era, such as the smart meter privacy problem, the agent identity of a control system is privacy-sensitive. When the agent identity is leaked, an adversary can further employ the corresponding optimal attacks on the control system.

### 2.2. Content and Contribution

We model the agent identity privacy problem as an adversarial binary hypothesis testing and employ the Kullback–Leibler divergence between the probability distributions of environment state sequences under different hypotheses as the privacy risk measure. We formulate a novel optimization problem and study the optimal privacy-preserving LQG policy. This work is compared with the previous research on privacy problems in Table 1.

The rest of this paper is organized as follows. In Section 3, we formulate the agent identity privacy problem in the LQG control system. In Section 4, we optimize the deterministic privacy-preserving LQG policy and give a sufficient condition for time-invariant optimal deterministic policy in the asymptotic regime. In Section 5, we discuss the random privacy-preserving LQG policy and show that the optimal linear Gaussian random policy reduces to the optimal deterministic privacy-preserving LQG policy. In Section 6, we present and analyze the numerical experiment results. Section 7 concludes this paper.

### 2.3. Notation

Unless otherwise specified, we denote a random scalar by a capital letter, e.g., *X*, its realization by the corresponding lower case letter, e.g., *x*, the Gaussian distribution with mean μ and variance $\sigma^{2}$ by $N(\mu ,\sigma^{2})$, the expectation operation by E(·), the Kullback–Leibler divergence between two probability distributions by D(·||·), and the natural logarithm by log(·).

## 3. Agent Identity Privacy Problem in LQG Control

**Figure 1:**
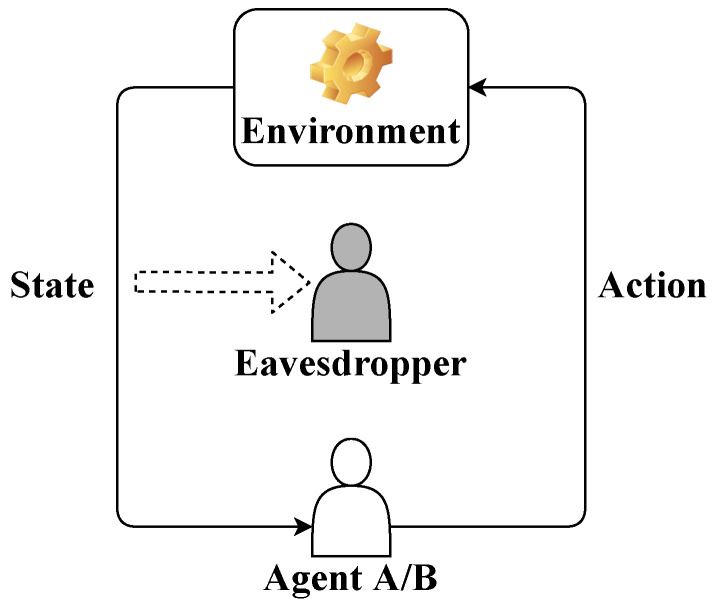
LQG control in the presence of an eavesdropper.

We consider an *N*-step LQG control in the presence of an eavesdropper as shown in Figure 1. There are two possible agents, Agent A and Agent B, which are with respect to a hypothesis H=0 and an alternative hypothesis H=1. We assume that the agents and the eavesdropper have perfect observations of the environment states. Based on the intercepted state sequence, the eavesdropper makes a binary hypothesis testing (A binary hypothesis is considered in this paper for simplification and can be extended to a multi-hypothesis.) to identify the current agent, which results in an agent identity privacy problem. To have a better understanding of the privacy problem, we give an example in the emerging application of autonomous vehicle. An autonomous vehicle can be controlled by a human driver (Agent A) or an autonomous driving system (Agent B). An adversary, who can be a compromised manager of the vehicle to everything (V2X) network, has access to the sensing data (environment state) of the autonomous vehicle and aims to attack the autonomous vehicle, e.g., to mislead the autonomous vehicle off the lane. To this end, the adversary needs to first identify if the current driver is the autonomous driving system by the intercepted sensing data sequence. The agent identity privacy problem commonly exists in intelligent autonomous systems, e.g., unmanned aerial vehicles and robots, where the autonomous control agents depending strongly on the sensing data are vulnerable to injection attacks and therefore the agent identities are privacy-sensitive.

The LQG control model for each agent is given as follows: For H=0 or H=1, 1≤i≤N,

(1) $$\begin{array}{c} s_{i+1}^{\left(H\right)}=\alpha s_{i}^{\left(H\right)}+\beta a_{i}^{\left(H\right)}+z_{i}, \end{array}$$

(2) $$\begin{array}{c} a_{i}^{\left(H\right)}=F_{i}^{\left(H\right)}\left(s_{i}^{\left(H\right)}\right), \end{array}$$

(3) $$\begin{array}{c} r_{i}^{\left(H\right)}=R^{\left(H\right)}\left(s_{i}^{\left(H\right)},a_{i}^{\left(H\right)}\right)=-\theta^{\left(H\right)}{\left(s_{i}^{\left(H\right)}\right)}^{2}-\phi^{\left(H\right)}{\left(a_{i}^{\left(H\right)}\right)}^{2}, \end{array}$$

(4) $$\begin{array}{c} S_{1}^{\left(H\right)}∼b_{1}^{\left(H\right)}≜N\left(\mu_{1},\sigma_{1}^{2}\right), \end{array}$$

(5) $$\begin{array}{c} Z_{i}∼N\left(0,\omega^{2}\right), \end{array}$$

where the parameters α≠0, β≠0, $\theta^{\left(H\right)}>0$, $\phi^{\left(H\right)}>0$, $\mu_{1}$, $\sigma_{1}^{2}>0$, and $\omega^{2}>0$ are given. The initial environment state $s_{1}^{\left(H\right)}$ is randomly generated following an *independent* Gaussian distribution. In the *i*-th time step, on observing the environment state $s_{i}^{\left(H\right)}$, the agent with respect to the hypothesis *H* employs the control policy $F_{i}^{\left(H\right)}$ to (randomly) determine an action $a_{i}^{\left(H\right)}$ as (2); the instantaneous control reward $r_{i}^{\left(H\right)}$ is jointly determined by the current state $s_{i}^{\left(H\right)}$ and action $a_{i}^{\left(H\right)}$ as (3); the next state $s_{i+1}^{\left(H\right)}$ is jointly determined by the current state $s_{i}^{\left(H\right)}$, the current action $a_{i}^{\left(H\right)}$, and $z_{i}$ randomly generated following an *independent* zero-mean Gaussian distribution as (1). In the standard LQG problem, the agent with respect to the hypothesis *H* only aims to maximize the expected accumulative reward by optimizing the control policies $F_{1:N}^{\left(H\right)}$:

(6) $$\begin{array}{c} F_{1:N}^{(H)*}=\arg\max_{F_{1:N}^{\left(H\right)}}E\left(\sum_{i=1}^{N}R^{\left(H\right)}\left(S_{i}^{\left(H\right)},A_{i}^{\left(H\right)}\right)\right). \end{array}$$

The optimal LQG control policy has been well established [35] and can be described as follows. For H=0 or H=1, 1≤i≤N,

(7) $$\begin{array}{c} {\tilde{\theta }}_{N+1}^{\left(H\right)}=0, \end{array}$$

(8) $$\begin{array}{c} {\tilde{\theta }}_{i}^{\left(H\right)}=L^{\left(H\right)}\left({\tilde{\theta }}_{i+1}^{\left(H\right)}\right)=\theta^{\left(H\right)}+{\tilde{\theta }}_{i+1}^{\left(H\right)}\alpha^{2}-\frac{{\left({\tilde{\theta }}_{i+1}^{\left(H\right)}\right)}^{2}\alpha^{2}\beta^{2}}{\phi^{\left(H\right)}+{\tilde{\theta }}_{i+1}^{\left(H\right)}\beta^{2}}>0, \end{array}$$

(9) $$\begin{array}{c} \kappa_{i}^{(H)*}=-\frac{{\tilde{\theta }}_{i+1}^{\left(H\right)}\alpha \beta }{\phi^{\left(H\right)}+{\tilde{\theta }}_{i+1}^{\left(H\right)}\beta^{2}}, \end{array}$$

(10) $$\begin{array}{c} F_{i}^{(H)*}\left(s_{i}^{\left(H\right)}\right)=\kappa_{i}^{(H)*}s_{i}^{\left(H\right)}. \end{array}$$

For H=0 or 1, it can be easily verified that the mapping $L^{\left(H\right)}$ is order-preserving, i.e., $L^{\left(H\right)}\left(x\right)\leq L^{H}\left(x^{\prime }\right)$ if $0\leq x\leq x^{\prime }$. From the Kleene’s fixed point theorem [36], it follows that

(11) $$\begin{array}{cc} {\tilde{\theta }}^{\left(H\right)}= & \;\lim_{N\to \infty }\underset{N\;iterations}{\underset{︸}{L^{\left(H\right)}(L^{\left(H\right)}(\cdots (L^{\left(H\right)}(L^{\left(H\right)}}}\left({\tilde{\theta }}_{N+1}^{\left(H\right)}\right)))\cdots ))\\ = & \;\theta^{\left(H\right)}+{\tilde{\theta }}^{\left(H\right)}\alpha^{2}-\frac{{\left({\tilde{\theta }}^{\left(H\right)}\right)}^{2}\alpha^{2}\beta^{2}}{\phi^{\left(H\right)}+{\tilde{\theta }}^{\left(H\right)}\beta^{2}}\\ = & \;\frac{\sqrt{{\left(\phi^{\left(H\right)}-\theta^{\left(H\right)}\beta^{2}-\phi^{\left(H\right)}\alpha^{2}\right)}^{2}+4\theta^{\left(H\right)}\phi^{\left(H\right)}\beta^{2}}-\left(\phi^{\left(H\right)}-\theta^{\left(H\right)}\beta^{2}-\phi^{\left(H\right)}\alpha^{2}\right)}{2\beta^{2}}. \end{array}$$

Therefore, if we consider the asymptotic regime as N→∞, the optimal control polices are *time-invariant*: For H=0 or H=1, i≥1,

(12) $$\begin{array}{c} \kappa^{(H)*}=-\frac{{\tilde{\theta }}^{\left(H\right)}\alpha \beta }{\phi^{\left(H\right)}+{\tilde{\theta }}^{\left(H\right)}\beta^{2}}, \end{array}$$

(13) $$\begin{array}{c} F_{i}^{(H)*}\left(s_{i}^{\left(H\right)}\right)=\kappa^{(H)*}s_{i}^{\left(H\right)}. \end{array}$$

For the agent identity privacy problem, we assume that the eavesdropper collects a sequence of environment states and carries out a binary hypothesis testing on the agent identity. Thus, the privacy risk can be measured by the hypothesis testing performance. In information theory, Kullback–Leibler divergence measures the “distance” between two probability distributions. When the value of the Kullback–Leibler divergence $D\left(p_{S_{1:N}^{\left(1\right)}}||p_{S_{1:N}^{\left(0\right)}}\right)$ is smaller, the random environment state sequences $S_{1:N}^{\left(0\right)}$ and $S_{1:N}^{\left(1\right)}$ are statistically “closer” to each other and it is more difficult for the eavesdropper to identify the current agent, i.e., a poorer hypothesis testing performance and a lower privacy risk. In this paper, we employ the Kullback–Leibler divergence $D\left(p_{S_{1:N}^{\left(1\right)}}||p_{S_{1:N}^{\left(0\right)}}\right)$ as the privacy risk measure.

Furthermore, we assume that both agents aim to improve their own expected accumulative rewards while only Agent B considers to reduce the privacy risk. This assumption makes sense in a lot of scenarios. In the aforementioned autonomous vehicle example, Agent A denotes the human driver and does not need to change the optimal driving style; Agent B denotes the autonomous driving system and can be reconfigured with respect to the human’s optimal driving style to improve the driving efficiency and to reduce the privacy risk. Under the assumption, Agent A takes the optimal LQG control policy as described by (7)–(10) with H=0. In the following, we focus on the privacy-preserving LQG control policy of Agent B. Taking into account the two design objectives of Agent B, we formulate the following optimization problem:

(14) $$\begin{array}{c} F_{1:N}^{(1)★}=\arg\max_{F_{1:N}^{\left(1\right)}}E\left(\sum_{i=1}^{N}R^{\left(1\right)}\left(S_{i}^{\left(1\right)},A_{i}^{\left(1\right)}\right)\right)-\lambda D\left(p_{S_{1:N}^{\left(1\right)}}||p_{S_{1:N}^{(0)*}}\right), \end{array}$$

where λ≥0 denotes the privacy-preserving design weight; the random environment state sequence $S_{1:N}^{(0)*}$ is induced by the optimal LQG policy $F_{1:N}^{(0)*}$ of Agent A. It follows from the chain rule of Kullback–Leibler divergence and the Markovian property of the state sequences that the privacy risk measure can be further decomposed as

(15) $$\begin{array}{cc} D\left(p_{S_{1:N}^{\left(1\right)}}||p_{S_{1:N}^{(0)*}}\right)= & \;D\left(p_{S_{1}^{\left(1\right)}}||p_{S_{1}^{\left(0\right)}}\right)+\sum_{i=2}^{N}D\left(p_{S_{i}^{\left(1\right)}|S_{i-1}^{\left(1\right)}}||p_{S_{i}^{(0)*}|S_{i-1}^{(0)*}}\right)\\ = & \;\sum_{i=2}^{N}D\left(p_{S_{i}^{\left(1\right)}|S_{i-1}^{\left(1\right)}}||p_{S_{i}^{(0)*}|S_{i-1}^{(0)*}}\right). \end{array}$$

It is obvious that the optimal privacy-preserving LQG control policy of Agent B depends on the value of λ. In the following two remarks, the optimal privacy-preserving LQG control policies are characterized for two special cases, λ=0 and λ→∞, respectively.

**Remark** **1.** *When λ=0, Agent B only aims to maximize the expected accumulative reward $E\left(\sum_{i=1}^{N}R^{\left(1\right)}\left(S_{i}^{\left(1\right)},A_{i}^{\left(1\right)}\right)\right)$. In this case, the optimal privacy-preserving LQG policy of Agent B reduces to the optimal LQG policy of Agent B, i.e., $F_{i}^{(1)★}\left(s_{i}^{\left(1\right)}\right)=F_{i}^{(1)*}\left(s_{i}^{\left(1\right)}\right)=\kappa_{i}^{(1)*}s_{i}^{\left(1\right)}$ for all 1≤i≤N.*

**Remark** **2.** *When λ→∞, Agent B only aims to minimize the privacy risk, which is measured by the Kullback–Leibler divergence $D\left(p_{S_{1:N}^{\left(1\right)}}||p_{S_{1:N}^{(0)*}}\right)$. In this case, the optimal privacy-preserving LQG policy of Agent B reduces to the optimal LQG policy of Agent A, i.e., $F_{i}^{(1)★}\left(s_{i}^{\left(1\right)}\right)=F_{i}^{(0)*}\left(s_{i}^{\left(1\right)}\right)=\kappa_{i}^{(0)*}s_{i}^{\left(1\right)}$ for all 1≤i≤N, and the minimum privacy risk is achieved, i.e., $D\left(p_{S_{1:N}^{(1)★}}||p_{S_{1:N}^{(0)*}}\right)=0$.*

When 0<λ<∞, we characterize the optimal privacy-preserving LQG control policies of Agent B in different forms in the following sections. For ease of reading, we list the parameters and their meanings in Table 2.

## 4. Deterministic Privacy-Preserving LQG Policy

When the privacy risk is not considered, as shown in (10), the optimal LQG control policy of Agent B is a deterministic linear mapping. In this section, we study the optimal deterministic privacy-preserving LQG policy of Agent B. Therefore, the policy of Agent B can be specified as: For 1≤i≤N,

(16) $$\begin{array}{c} F_{i}^{\left(1\right)}:R\to R. \end{array}$$

In the following theorem, we characterize the optimal deterministic privacy-preserving LQG policy of Agent B.

**Theorem** **1.** *At each step, the optimal deterministic privacy-preserving LQG policy of Agent B with respect to the optimization problem *(14)* is a linear mapping as: For 1≤i≤N,*

(17) $${\hat{\theta }}_{N+1}^{\left(1\right)}=0,$$

(18) $${\hat{\theta }}_{i}^{\left(1\right)}=J_{N+1-i}\left({\hat{\theta }}_{i+1}^{\left(1\right)}\right)=\theta^{\left(1\right)}+{\hat{\theta }}_{i+1}^{\left(1\right)}\alpha^{2}+\frac{\lambda }{2\omega^{2}}\beta^{2}{\left(\kappa_{i}^{(0)*}\right)}^{2}-\frac{{\left(\frac{\lambda }{2\omega^{2}}\beta^{2}\kappa_{i}^{(0)*}-{\hat{\theta }}_{i+1}^{\left(1\right)}\alpha \beta \right)}^{2}}{\phi^{\left(1\right)}+{\hat{\theta }}_{i+1}^{\left(1\right)}\beta^{2}+\frac{\lambda }{2\omega^{2}}\beta^{2}}>0,$$

(19) $$\kappa_{i}^{(1)★}=\frac{\frac{\lambda }{2\omega^{2}}\beta^{2}\kappa_{i}^{(0)*}-{\hat{\theta }}_{i+1}^{\left(1\right)}\alpha \beta }{\phi^{\left(1\right)}+{\hat{\theta }}_{i+1}^{\left(1\right)}\beta^{2}+\frac{\lambda }{2\omega^{2}}\beta^{2}},$$

(20) $$F_{i}^{(1)★}\left(s_{i}^{\left(1\right)}\right)=\kappa_{i}^{(1)★}s_{i}^{\left(1\right)}.$$

*Then, the maximum achievable weighted design objective of Agent B is*

(21) $$\begin{array}{c} \max_{F_{1:N}^{\left(1\right)}}E\left(\sum_{i=1}^{N}R^{\left(1\right)}\left(S_{i}^{\left(1\right)},A_{i}^{\left(1\right)}\right)\right)-\lambda D\left(p_{S_{1:N}^{\left(1\right)}}||p_{S_{1:N}^{(0)*}}\right)=-{\hat{\theta }}_{1}^{\left(1\right)}\left(\mu_{1}^{2}+\sigma_{1}^{2}\right)-\omega^{2}\sum_{i=1}^{N-1}{\hat{\theta }}_{i+1}^{\left(1\right)}. \end{array}$$

The proof of Theorem 1 is presented in Appendix A.

**Remark** **3.** *When λ=0, it is easy to show that $\kappa_{i}^{(1)★}=\kappa_{i}^{(1)*}$ for all 1≤i≤N, i.e., the optimal deterministic privacy-preserving LQG policy is consistent with the optimal privacy-preserving LQG policy shown in Remark 1.*

**Remark** **4.** *It is easy to show that $\lim_{\lambda \to \infty }\kappa_{i}^{(1)★}=\kappa_{i}^{(0)*}$ for all 1≤i≤N, i.e., the optimal deterministic privacy-preserving LQG policy is consistent with the optimal privacy-preserving LQG policy shown in Remark 2.*

**Remark** **5.** *Although the objective in (14) is a linear combination of the expected accumulative reward and the privacy risk measured by the Kullback–Leibler divergence, the optimal linear coefficient $\kappa_{i}^{(1)★}$ is a non-linear function of $\kappa_{i}^{(1)*}$ (the optimal linear coefficient with respect to only maximize the expected accumulative reward) and $\kappa_{i}^{(0)*}$ (the optimal linear coefficient with respect to only minimize the privacy risk) when we consider the deterministic privacy-preserving LQG control policy of Agent B.*

**Remark** **6.** *When Agent B employs the optimal deterministic privacy-preserving LQG policy at each step, the random state-action sequence is jointly Gaussian distributed.*

In the asymptotic regime as N→∞, the optimal LQG control policy is time-invariant. In this case, the design of the optimal policy becomes an easier task. Theorem 2 gives a sufficient condition such that the optimal deterministic privacy-preserving LQG policy of Agent B is time-invariant in the asymptotic regime.

**Theorem** **2.** *When the model parameters satisfy the following inequality*

(22) $$\begin{array}{c} \frac{\left|\frac{\lambda }{2\omega^{2}}\beta^{4}{\left(\kappa^{(0)*}\right)}^{2}\phi^{\left(1\right)}-\left(\phi^{\left(1\right)}\alpha^{2}+\frac{\lambda }{2\omega^{2}}\beta^{2}{\left(\alpha +\beta \kappa^{(0)*}\right)}^{2}\right)\left(\phi^{\left(1\right)}+\frac{\lambda }{2\omega^{2}}\beta^{2}\right)\right|}{{\left(\phi^{\left(1\right)}+\frac{\lambda }{2\omega^{2}}\beta^{2}\right)}^{2}}<1, \end{array}$$

*the optimal deterministic privacy-preserving LQG policy of Agent B is time-invariant in the asymptotic regime. More specifically, $J_{N}\left(J_{N-1}\left(\cdots \left(J_{2}\left(J_{1}\left({\hat{\theta }}_{N+1}^{\left(1\right)}\right)\right)\right)\cdots \right)\right)$ converges to the unique fixed point ${\hat{\theta }}^{\left(1\right)}$ as*

(23) $$\begin{array}{cc} {\hat{\theta }}^{\left(1\right)}= & \;\lim_{N\to \infty }J_{N}\left(J_{N-1}\left(\cdots \left(J_{2}\left(J_{1}\left({\hat{\theta }}_{N+1}^{\left(1\right)}\right)\right)\right)\cdots \right)\right)\\ = & \;\theta^{\left(1\right)}+\alpha^{2}{\hat{\theta }}^{\left(1\right)}+\frac{\lambda }{2\omega^{2}}\beta^{2}{\left(\kappa^{(0)*}\right)}^{2}-\frac{{\left(\frac{\lambda }{2\omega^{2}}\beta^{2}\kappa^{(0)*}-\alpha \beta {\hat{\theta }}^{\left(1\right)}\right)}^{2}}{\phi^{\left(1\right)}+\beta^{2}{\hat{\theta }}^{\left(1\right)}+\frac{\lambda }{2\omega^{2}}\beta^{2}}; \end{array}$$

*and the time-invariant optimal deterministic privacy-preserving LQG policy of Agent B can be described by*

(24) $$\begin{array}{c} \kappa^{(1)★}=\frac{\frac{\lambda }{2\omega^{2}}\beta^{2}\kappa^{(0)*}-{\hat{\theta }}^{\left(1\right)}\alpha \beta }{\phi^{\left(1\right)}+{\hat{\theta }}^{\left(1\right)}\beta^{2}+\frac{\lambda }{2\omega^{2}}\beta^{2}}, \end{array}$$

(25) $$\begin{array}{c} F_{i}^{(1)★}\left(s_{i}^{\left(1\right)}\right)=\kappa^{(1)★}s_{i}^{\left(1\right)}. \end{array}$$

*Under this condition, the asymptotic weighted design object rate of Agent B achieved by the time-invariant optimal deterministic privacy-preserving LQG policy is*

(26) $$\begin{array}{c} \lim_{N\to \infty }\frac{1}{N}\max_{F_{1:N}^{\left(1\right)}}E\left(\sum_{i=1}^{N}R^{\left(1\right)}\left(S_{i}^{\left(1\right)},A_{i}^{\left(1\right)}\right)\right)-\lambda D\left(p_{S_{1:N}^{\left(1\right)}}||p_{S_{1:N}^{(0)*}}\right)=-\omega^{2}{\hat{\theta }}^{\left(1\right)}. \end{array}$$

The proof of Theorem 2 is given in Appendix B.

## 5. Random Privacy-Preserving LQG Policy

As shown in Theorem 1, the optimal deterministic privacy-preserving LQG policy of Agent B is a linear mapping. In this section, we first discuss the optimal random privacy-preserving LQG policy and then consider a particular random policy by extending the deterministic linear mapping to the linear Gaussian random policy for Agent B. Here, the random policy of Agent B can be specified as: For 1≤i≤N,

(27) $$\begin{array}{c} F_{i}^{\left(1\right)}:R\times R\to R_{\geq 0}. \end{array}$$

With slight abuse of notation, we denote the condition probability (density) of taking the action $a_{i}^{\left(1\right)}\in R$ given the state $s_{i}^{\left(1\right)}\in R$ and the random policy $F_{i}^{\left(1\right)}$ by $F_{i}^{\left(1\right)}\left(a_{i}^{\left(1\right)}\left|s_{i}^{\left(1\right)}\right.\right)\in R_{\geq 0}$.

It can be easily shown that the optimal random privacy-preserving LQG policy of Agent B in the final step $F_{N}^{(1)★}$ reduces to the deterministic linear mapping in (A2). For 1≤i≤N−1, it follows from the backward dynamic programming that the optimal random privacy-preserving LQG policy of Agent B in the *i*-th step does not reduce to a deterministic linear mapping in general. That is because the conditional probability distribution $p_{S_{i+1}^{\left(1\right)}|S_{i}^{\left(1\right)}}$ given a random policy $F_{i}^{\left(1\right)}$ is a Gaussian mixture model and then the Kullback–Leibler divergence $D\left(p_{S_{i+1}^{\left(1\right)}|S_{i}^{\left(1\right)}}||p_{S_{i+1}^{(0)*}|S_{i}^{(0)*}}\right)$ between a Gaussian mixture model and a Gaussian distribution generally does not reduce to the quadratic mean of $A_{i}^{\left(1\right)}-\kappa_{i}^{(0)*}S_{i}^{\left(1\right)}$ as (A5). To the best of our knowledge, there is no analytically tractable formula for Kullback–Leibler divergence between Gaussian mixture models and only approximations are available [37,38,39]. Therefore, we do not give the close-form solution of the optimal random privacy-preserving LQG policy in this paper.

In what follows, we focus on the linear Gaussian random policy: For 1≤i≤N,

(28) $$\begin{array}{c} F_{i}^{\left(1\right)}\left(s_{i}^{\left(1\right)}\right)=\kappa_{i}^{\left(1\right)}s_{i}^{\left(1\right)}+w_{i}^{\left(1\right)}, \end{array}$$

where $w_{i}^{\left(1\right)}$ is the realization of an *independent* zero-mean Gaussian random process noise $W_{i}^{\left(1\right)}∼N\left(0,\delta_{i}^{2}\right)$. Thus, a linear Gaussian random policy $F_{i}^{\left(1\right)}$ can be completely described by the parameters $\left(\kappa_{i}^{\left(1\right)},\delta_{i}^{2}\right)$. Theorem 3 characterizes the optimal linear Gaussian random privacy-preserving LQG policy of Agent B.

**Theorem** **3.** *At each step, the optimal linear Gaussian random privacy-preserving LQG policy of Agent B with respect to the optimization problem *(14)* is the same deterministic linear mapping as in Theorem 1.*

The proof of Theorem 3 is presented in Appendix C.

**Remark** **7.** *Adding an independent zero-mean Gaussian random process noise to the linear mapping of the optimal deterministic privacy-preserving LQG policy cannot improve the performance of Agent B.*

## 6. Numerical Experiments

### 6.1. Convergence of the Sequence $\left({\hat{\theta }}_{N+1}^{\left(1\right)},{\hat{\theta }}_{N}^{\left(1\right)},{\hat{\theta }}_{N-1}^{\left(1\right)},\cdots \right)$

**Figure 2:**
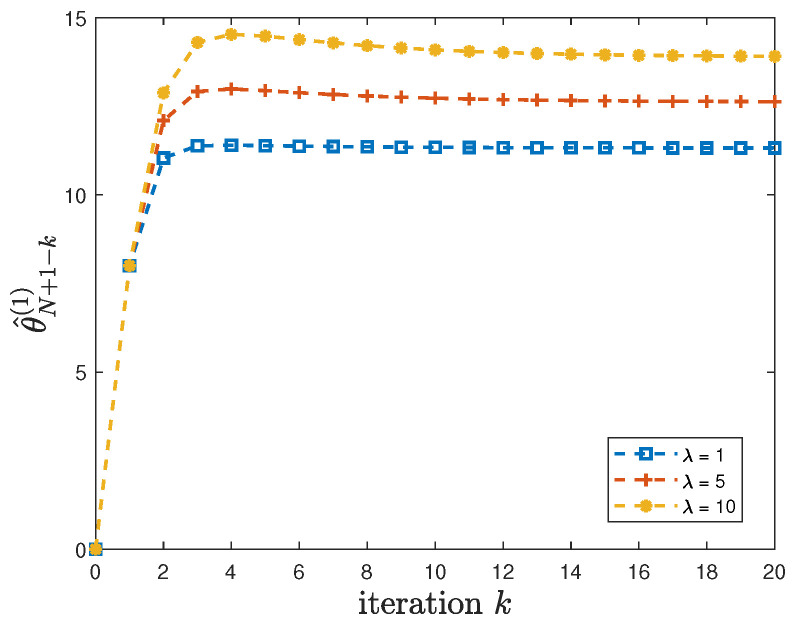
For λ=1, 5 or 10, the convergence of ${\hat{\theta }}_{N+1-k}^{\left(1\right)}=J_{k}\left(J_{k-1}\left(\cdots \left(J_{2}\left(J_{1}\left({\hat{\theta }}_{N+1}^{\left(1\right)}\right)\right)\right)\cdots \right)\right)$.

When the constraint (22) in Theorem 2 is satisfied, we first illustrate the convergence of the sequence $\left({\hat{\theta }}_{N+1}^{\left(1\right)},{\hat{\theta }}_{N}^{\left(1\right)},{\hat{\theta }}_{N-1}^{\left(1\right)},\cdots \right)$. In addition to the default model parameters in Table 3, we set $\theta^{\left(1\right)}=8$, $\phi^{\left(1\right)}=1$, and let the privacy-preserving design weight λ=1, 5 or 10. By using these parameters, it can be easily verified that the constraint (22) is satisfied. Figure 2 shows that ${\hat{\theta }}_{N+1-k}^{\left(1\right)}=J_{k}\left(J_{k-1}\left(\cdots \left(J_{2}\left(J_{1}\left({\hat{\theta }}_{N+1}^{\left(1\right)}\right)\right)\right)\cdots \right)\right)$ converges after k=20 iterations for different values of λ. Furthermore, different convergence patterns can be observed for different values of λ.

### 6.2. Impact of the Privacy-Preserving Design Weight λ

**Figure 3:**
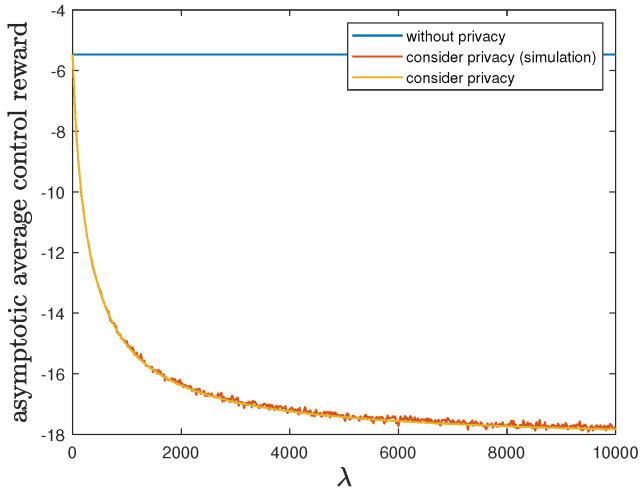
When 0≤λ≤ 10,000, comparison of the asymptotic average control reward $\lim_{N\to \infty }\frac{1}{N}E\left(\sum_{i=1}^{N}R^{\left(1\right)}\left(S_{i}^{(1)*},A_{i}^{(1)*}\right)\right)$ achieved by the time-invariant optimal LQG policy of Agent B and the asymptotic average control reward $\lim_{N\to \infty }\frac{1}{N}E\left(\sum_{i=1}^{N}R^{\left(1\right)}\left(S_{i}^{(1)★},A_{i}^{(1)★}\right)\right)$ achieved by the time-invariant optimal deterministic privacy-preserving LQG policy of Agent B.

**Figure 4:**
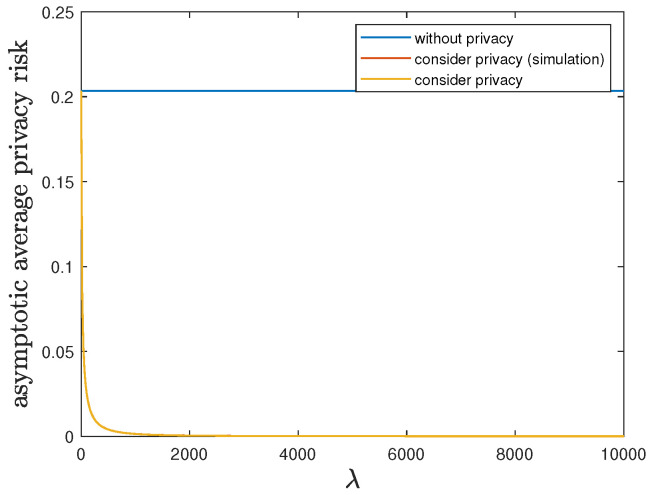
When 0≤λ≤ 10,000, comparison of the asymptotic average privacy risk $\lim_{N\to \infty }\frac{1}{N}D\left(p_{S_{1:N}^{(1)*}}||p_{S_{1:N}^{(0)*}}\right)$ achieved by the time-invariant optimal LQG policy of Agent B and the asymptotic average privacy risk $\lim_{N\to \infty }\frac{1}{N}D\left(p_{S_{1:N}^{(1)★}}||p_{S_{1:N}^{(0)*}}\right)$ achieved by the time-invariant optimal deterministic privacy-preserving LQG policy of Agent B.

Here, we show the impact of the privacy-preserving design weight λ on the trade-off between the control reward of Agent B and the privacy risk. We use the same parameters as in Section 6.1, but allow 0≤λ≤ 10,000. Then, Theorem 2 is applicable and therefore the optimal deterministic privacy-preserving LQG policy of Agent B is time-invariant in the asymptotic regime. Figure 3 and Figure 4 show that both the asymptotic average control reward $\lim_{N\to \infty }\frac{1}{N}E\left(\sum_{i=1}^{N}R^{\left(1\right)}\left(S_{i}^{(1)★},A_{i}^{(1)★}\right)\right)$ and the asymptotic average privacy risk $\lim_{N\to \infty }\frac{1}{N}D\left(p_{S_{1:N}^{(1)★}}||p_{S_{1:N}^{(0)*}}\right)$ achieved by the time-invariant optimal deterministic privacy-preserving LQG policy of Agent B decrease as λ increases, i.e., the control reward of Agent B is degraded while the privacy is enhanced. When the privacy risk is not considered, the best control reward of Agent B is achieved at the cost of the highest privacy risk.

In addition to the analytical results, we also present the simulation results by considering privacy in Figure 3 and Figure 4. Given 0≤λ≤ 10,000, we employ the corresponding time-invariant optimal deterministic privacy-preserving LQG policy of Agent B and run the 10,000-step privacy-preserving LQG control with 100 randomly generated initial states. Then, the average control reward and the average privacy risk are evaluated and compared with the analytical results of asymptotic average control reward and asymptotic average privacy risk, respectively. As shown in Figure 3 and Figure 4, the simulation results match quite well with the analytical results, which validates our analytical results.

### 6.3. Impact of Parameter $\theta^{\left(1\right)}$

**Figure 5:**
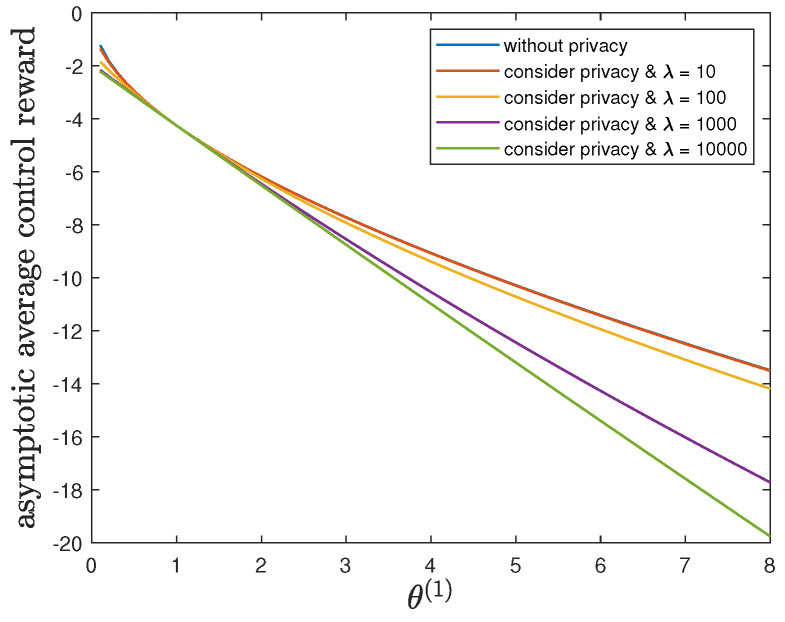
For $0.01\leq \theta^{\left(1\right)}\leq 8$ and λ=0 (without privacy), 10, 100, 1000 or 10,000, comparison of the asymptotic average control reward $\lim_{N\to \infty }\frac{1}{N}E\left(\sum_{i=1}^{N}R^{\left(1\right)}\left(S_{i}^{(1)*},A_{i}^{(1)*}\right)\right)$ achieved by the time-invariant optimal LQG policy of Agent B and the asymptotic average control reward $\lim_{N\to \infty }\frac{1}{N}E\left(\sum_{i=1}^{N}R^{\left(1\right)}\left(S_{i}^{(1)★},A_{i}^{(1)★}\right)\right)$ achieved by the time-invariant optimal deterministic privacy-preserving LQG policy of Agent B.

**Figure 6:**
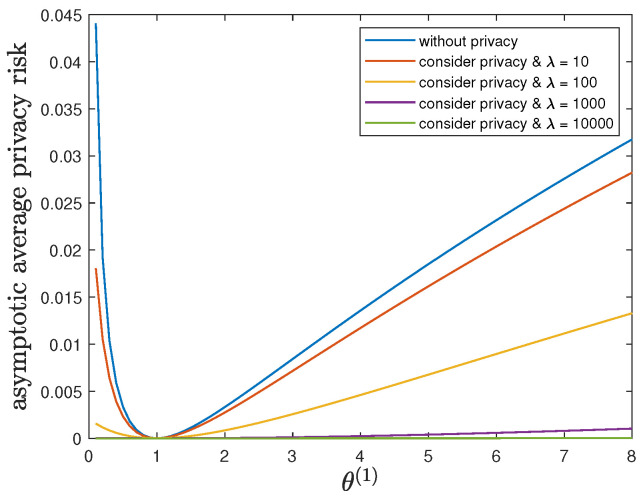
For $0.01\leq \theta^{\left(1\right)}\leq 8$ and λ=0 (without privacy), 10, 100, 1000 or 10,000, comparison of the asymptotic average privacy risk $\lim_{N\to \infty }\frac{1}{N}D\left(p_{S_{1:N}^{(1)*}}||p_{S_{1:N}^{(0)*}}\right)$ achieved by the time-invariant optimal LQG policy of Agent B and the asymptotic average privacy risk $\lim_{N\to \infty }\frac{1}{N}D\left(p_{S_{1:N}^{(1)★}}||p_{S_{1:N}^{(0)*}}\right)$ achieved by the time-invariant optimal deterministic privacy-preserving LQG policy of Agent B.

Here, we study the impact of the parameter $\theta^{\left(1\right)}$ on the control reward of Agent B and the privacy risk. In addition to the default model parameters in Table 3, we set $\phi^{\left(1\right)}=\phi^{\left(0\right)}=16$ and allow $0.01\leq \theta^{\left(1\right)}\leq 8$, λ=0 (without privacy), 10, 100, 1000 or 10,000. It can be verified that Theorem 2 holds for those model parameters. For all $0.01\leq \theta^{\left(1\right)}\leq 8$ and by increasing the value of λ, Figure 5 and Figure 6 show a trade-off between the control reward of Agent B and the privacy risk, which is consistent with the previous observations. For λ=0, 10, 100, 1000 or 10,000, Figure 5 shows that the asymptotic average control reward of Agent B decreases as $\theta^{\left(1\right)}$ increases. This is reasonable since $-\theta^{\left(1\right)}$ is the quadratic coefficient in the instantaneous reward function $R^{\left(1\right)}$. For λ=0, 10, 100, 1000 or 10,000, Figure 6 shows that the asymptotic average privacy risk has a pattern to decrease first, then to increase, and to achieve the minimum value 0 when $\theta^{\left(1\right)}=\theta^{\left(0\right)}=1$. When $\theta^{\left(1\right)}=\theta^{\left(0\right)}=1$, both agents have the same instantaneous reward function and employ the same optimal LQG control policy, which leads to the same state sequence distribution under both hypotheses and the minimum value 0 of the Kullback–Leibler divergence. As $\theta^{\left(1\right)}$ deviates from the value of $\theta^{\left(0\right)}$, the agents have more different instantaneous reward functions, which lead to more different state sequence distributions under both hypotheses and a larger value of the Kullback–Leibler divergence.

### 6.4. Impact of Parameter $\phi^{\left(1\right)}$

**Figure 7:**
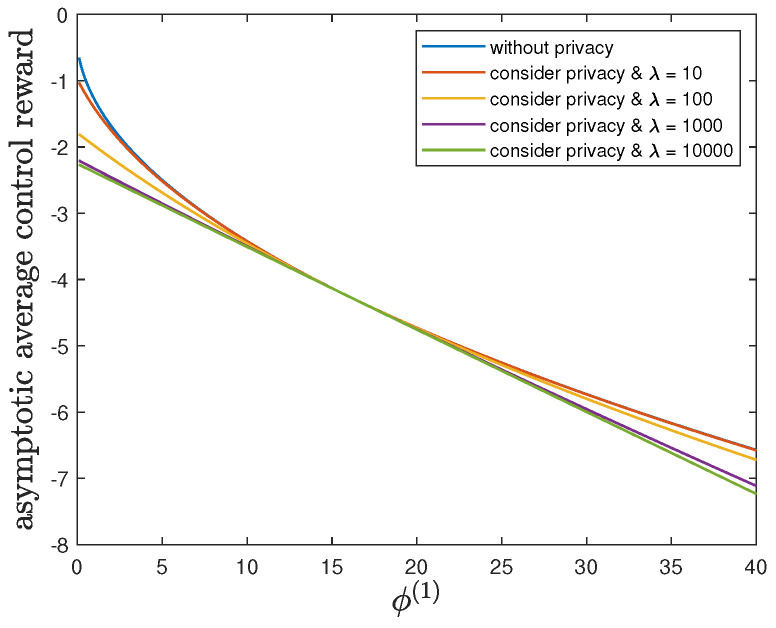
For $0.01\leq \phi^{\left(1\right)}\leq 40$ and λ=0 (without privacy), 10, 100, 1000 or 10,000, comparison of the asymptotic average control reward $\lim_{N\to \infty }\frac{1}{N}E\left(\sum_{i=1}^{N}R^{\left(1\right)}\left(S_{i}^{(1)*},A_{i}^{(1)*}\right)\right)$ achieved by the time-invariant optimal LQG policy of Agent B and the asymptotic average control reward $\lim_{N\to \infty }\frac{1}{N}E\left(\sum_{i=1}^{N}R^{\left(1\right)}\left(S_{i}^{(1)★},A_{i}^{(1)★}\right)\right)$ achieved by the time-invariant optimal deterministic privacy-preserving LQG policy of Agent B.

**Figure 8:**
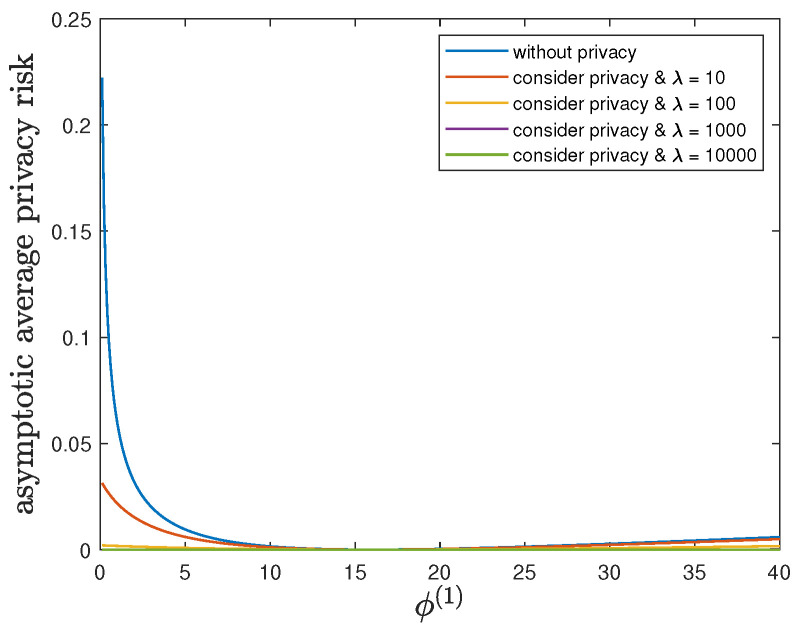
For $0.01\leq \phi^{\left(1\right)}\leq 40$ and λ=0 (without privacy), 10, 100, 1000 or 10,000, comparison of the asymptotic average privacy risk $\lim_{N\to \infty }\frac{1}{N}D\left(p_{S_{1:N}^{(1)*}}||p_{S_{1:N}^{(0)*}}\right)$ achieved by the time-invariant optimal LQG policy of Agent B and the asymptotic average privacy risk $\lim_{N\to \infty }\frac{1}{N}D\left(p_{S_{1:N}^{(1)★}}||p_{S_{1:N}^{(0)*}}\right)$ achieved by the time-invariant optimal deterministic privacy-preserving LQG policy of Agent B.

Here, we show the impact of the parameter $\phi^{\left(1\right)}$ on the control reward of Agent B and the privacy risk. In addition to the default model parameters in Table 3, we set $\theta^{\left(1\right)}=\theta^{\left(0\right)}=1$ and allow $0.01\leq \phi^{\left(1\right)}\leq 40$, λ=0 (without privacy), 10, 100, 1000 or 10,000. It can be verified that Theorem 2 holds for those model parameters. For all $0.01\leq \phi^{\left(1\right)}\leq 40$ and by increasing the value of λ, Figure 7 and Figure 8 also show a trade-off between the control reward of Agent B and the privacy risk. For λ=0, 10, 100, 1000 or 10,000, Figure 7 shows that the asymptotic average control reward of Agent B decreases as $\phi^{\left(1\right)}$ increases. This is because $-\phi^{\left(1\right)}$ is the other quadratic coefficient in the instantaneous reward function $R^{\left(1\right)}$. For λ=0, 10, 100, 1000 or 10,000, Figure 8 shows that the asymptotic average privacy risk has a similar pattern to decrease first, then to increase, and to achieve the minimum value 0 when $\phi^{\left(1\right)}=\phi^{\left(0\right)}=16$. This pattern can be similarly explained as Section 6.3.

### 6.5. Impact of Parameter $\theta^{\left(0\right)}$

**Figure 9:**
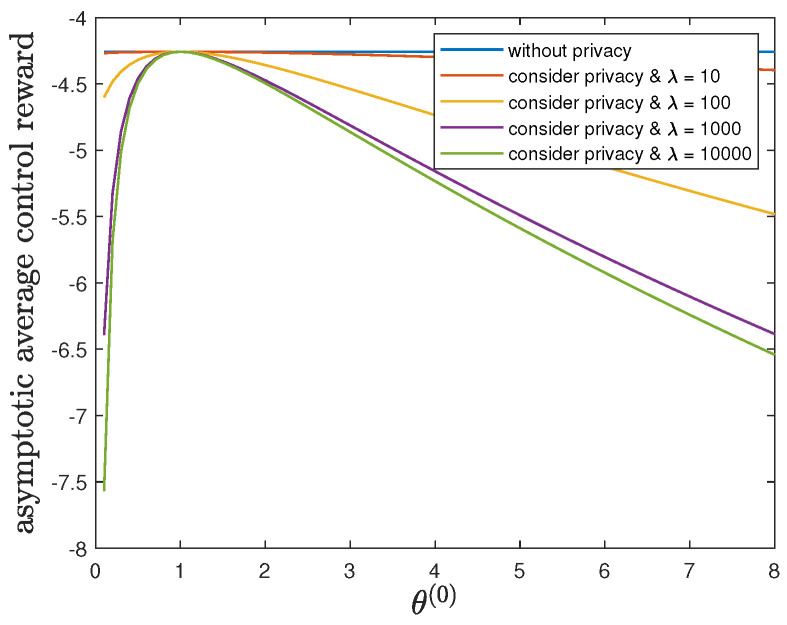
For $\theta^{\left(1\right)}=1$, $\phi^{\left(1\right)}=\phi^{\left(0\right)}=16$, $0.01\leq \theta^{\left(0\right)}\leq 8$, and λ=0 (without privacy), 10, 100, 1000 or 10,000, comparison of the asymptotic average control reward $\lim_{N\to \infty }\frac{1}{N}E\left(\sum_{i=1}^{N}R^{\left(1\right)}\left(S_{i}^{(1)*},A_{i}^{(1)*}\right)\right)$ achieved by the time-invariant optimal LQG policy of Agent B and the asymptotic average control reward $\lim_{N\to \infty }\frac{1}{N}E\left(\sum_{i=1}^{N}R^{\left(1\right)}\left(S_{i}^{(1)★},A_{i}^{(1)★}\right)\right)$ achieved by the time-invariant optimal deterministic privacy-preserving LQG policy of Agent B.

**Figure 10:**
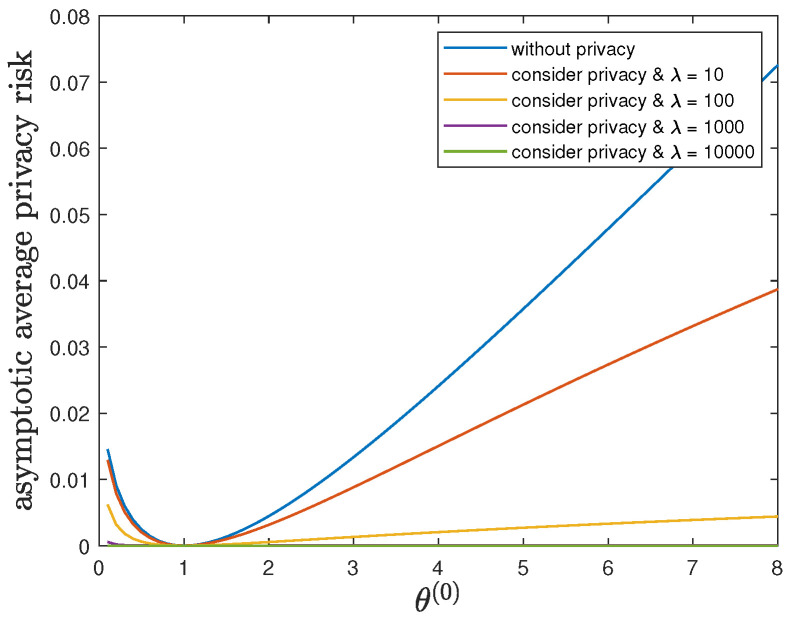
For $\theta^{\left(1\right)}=1$, $\phi^{\left(1\right)}=\phi^{\left(0\right)}=16$, $0.01\leq \theta^{\left(0\right)}\leq 8$, and λ=0 (without privacy), 10, 100, 1000 or 10,000, comparison of the asymptotic average privacy risk $\lim_{N\to \infty }\frac{1}{N}D\left(p_{S_{1:N}^{(1)*}}||p_{S_{1:N}^{(0)*}}\right)$ achieved by the time-invariant optimal LQG policy of Agent B and the asymptotic average privacy risk $\lim_{N\to \infty }\frac{1}{N}D\left(p_{S_{1:N}^{(1)★}}||p_{S_{1:N}^{(0)*}}\right)$ achieved by the time-invariant optimal deterministic privacy-preserving LQG policy of Agent B.

By fixing $\theta^{\left(1\right)}=1$ and $\phi^{\left(1\right)}=\phi^{\left(0\right)}=16$, we study the impact of the parameter $\theta^{\left(0\right)}$ on the control reward of Agent B and the privacy risk. In addition to the default model parameters in Table 3, we allow $0.01\leq \theta^{\left(0\right)}\leq 8$ and λ=0 (without privacy), 10, 100, 1000 or 10,000. It can be verified that Theorem 2 holds for those model parameters. For all $0.01\leq \theta^{\left(0\right)}\leq 8$ and by increasing the value of λ, Figure 9 and Figure 10 show a trade-off between the control reward of Agent B and the privacy risk. For λ=0, 10, 100, 1000 or 10,000, Figure 9 and Figure 10 show that the asymptotic average control reward of Agent B achieves the maximum value while the asymptotic average privacy risk achieves the minimum value 0 when $\theta^{\left(1\right)}=\theta^{\left(0\right)}=1$. In this case, both agents have the same instantaneous reward function and employ the same optimal LQG control policy, which maximizes their control rewards, leads to the same state sequence distribution under both hypotheses, and therefore achieves the minimum value 0 of the Kullback–Leibler divergence.

### 6.6. Impact of Parameter $\phi^{\left(0\right)}$

**Figure 11:**
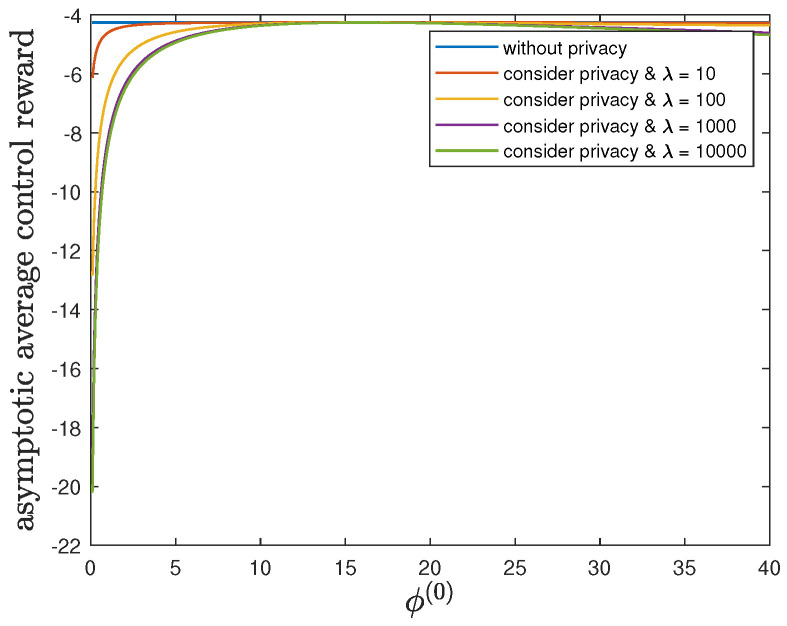
For $\theta^{\left(1\right)}=\theta^{\left(0\right)}=1$, $\phi^{\left(1\right)}=16$, $0.01\leq \phi^{\left(0\right)}\leq 40$, and λ=0 (without privacy), 10, 100, 1000 or 10,000, comparison of the asymptotic average control reward $\lim_{N\to \infty }\frac{1}{N}E\left(\sum_{i=1}^{N}R^{\left(1\right)}\left(S_{i}^{(1)*},A_{i}^{(1)*}\right)\right)$ achieved by the time-invariant optimal LQG policy of Agent B and the asymptotic average control reward $\lim_{N\to \infty }\frac{1}{N}E\left(\sum_{i=1}^{N}R^{\left(1\right)}\left(S_{i}^{(1)★},A_{i}^{(1)★}\right)\right)$ achieved by the time-invariant optimal deterministic privacy-preserving LQG policy of Agent B.

**Figure 12:**
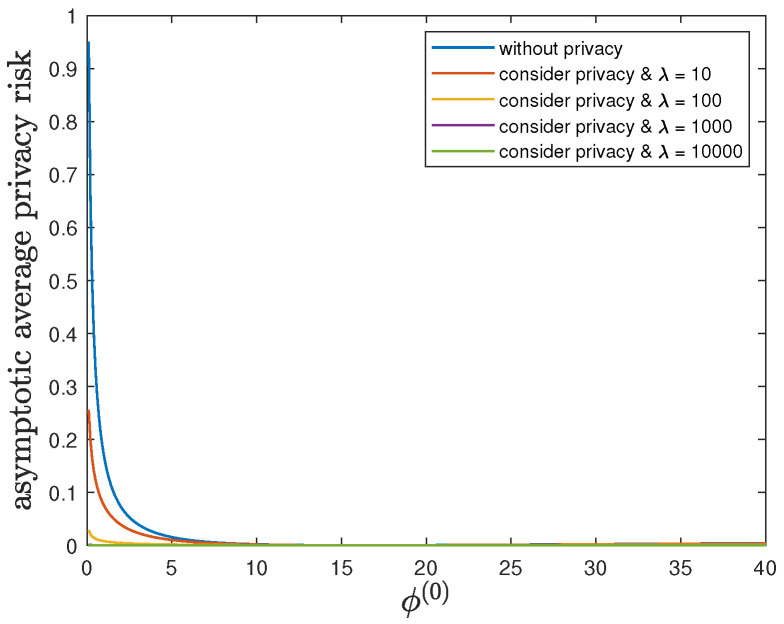
For $\theta^{\left(1\right)}=\theta^{\left(0\right)}=1$, $\phi^{\left(1\right)}=16$, $0.01\leq \phi^{\left(0\right)}\leq 40$, and λ=0 (without privacy), 10, 100, 1000 or 10,000, comparison of the asymptotic average privacy risk $\lim_{N\to \infty }\frac{1}{N}D\left(p_{S_{1:N}^{(1)*}}||p_{S_{1:N}^{(0)*}}\right)$ achieved by the time-invariant optimal LQG policy of Agent B and the asymptotic average privacy risk $\lim_{N\to \infty }\frac{1}{N}D\left(p_{S_{1:N}^{(1)★}}||p_{S_{1:N}^{(0)*}}\right)$ achieved by the time-invariant optimal deterministic privacy-preserving LQG policy of Agent B.

By fixing $\phi^{\left(1\right)}=16$ and $\theta^{\left(1\right)}=\theta^{\left(0\right)}=1$, we study the impact of the parameter $\phi^{\left(0\right)}$ on the control reward of Agent B and the privacy risk. In addition to the default model parameters in Table 3, we allow $0.01\leq \phi^{\left(0\right)}\leq 40$ and λ=0 (without privacy), 10, 100, 1000 or 10,000. From Figure 11 and Figure 12, we have similar observations of the impact of $\phi^{\left(0\right)}$ as in Section 6.5. These observations here can be similarly explained as well.

## 7. Conclusions

In this paper, we consider the agent identity privacy problem in the scalar LQG control. Regarding this novel privacy problem, we model it as an adversarial binary hypothesis testing and employ the Kullback–Leibler divergence to measure the privacy risk. We then formulate a novel privacy-preserving LQG control optimization by taking into account both the accumulative control reward of Agent B and the privacy risk. We prove that the optimal deterministic privacy-preserving LQG control policy of Agent B is a linear mapping, which is consistent with the standard LQG. We further show that the random policy formulated by adding an independent Gaussian random process noise to the optimal deterministic privacy-preserving LQG policy cannot improve the performance. We also give a sufficient condition to guarantee the time-invariant optimal deterministic privacy-preserving LQG policy in the asymptotic regime.

This research can be extended in our future works. Studying the general random policy of Agent B is an interesting extension. This theoretic study can be extended to develop privacy-preserving reinforcement learning algorithms. The problem can also be extended and formulated as a non-cooperative game of multiple agents with conflicting objectives, where some agents only aim to optimize their own accumulative control rewards while the other agents consider the agent identity privacy risk in addition to their own accumulative control rewards. 

## Figures and Tables

**Table 1:** Comparison of research on privacy problems.

	Private Information	Privacy Model/Measure	Privacy Mechanism
[26]	State	Equivocation	Privacy-preserving policy design
[27,28,29,30]	State	Differential privacy	Adding privacy noise to state
[32]	Reward function	Differential privacy	Adding privacy noise to value function
[33]	The whole LQG system	Computational secrecy	Labeled homomorphic encryption
This work	Agent identity	Kullback–Leibler divergence	Privacy-preserving policy design

**Table 2:** Parameters.

Parameter	Meaning	Parameter	Meaning
*N*	Number of steps	*H*	Agent identity binary hypothesis
α, β	Time-invariant linear coefficients in the linear Gaussian dynamic model	$z_{i}$, $\omega^{2}$	Independent zero-mean Gaussian-distributed disturbance noise in the *i*-th step and its variance
$s_{i}^{\left(H\right)}$	State of the agent (H) in the *i*-th step	$a_{i}^{\left(H\right)}$	Action of the agent (H) in the *i*-th step
$F_{i}^{\left(H\right)}$	Policy of the agent (H) in the *i*-th step	$\kappa_{i}^{\left(H\right)}$	State feedback gain of a linear policy of the agent (H) in the *i*-th step
$r_{i}^{\left(H\right)}$	Instantaneous control reward of the agent (H) in the *i*-th step	$R^{\left(H\right)}$, $\theta^{\left(H\right)}$, $\phi^{\left(H\right)}$	Time-invariant instantaneous quadratic control reward function of the agent (H) and its coefficients
$\mu_{1}$, $\sigma_{1}^{2}$	Mean and variance of the Gaussian-distributed initial state	λ	Privacy-preserving design weight

**Table 3:** Default model parameters.

Parameter	$\mu_{1}$	$\sigma_{1}^{2}$	α	β	$\omega^{2}$	$\theta^{\left(0\right)}$	$\phi^{\left(0\right)}$
**Value**	1	1	1	0.5	0.5	1	16

## Data Availability

Not applicable.

## Appendix A

**Proof** **of Theorem 1.** The proof is based on the backward dynamic programming. We first consider the sub-problem of the final step. Given a probability distribution $p_{S_{N}^{\left(1\right)}}$, the final step optimization problem of the deterministic control policy $F_{N}^{\left(1\right)}$ is

(A1) $$\begin{array}{cc} F_{N}^{(1)★}= & \;\arg\max_{F_{N}^{\left(1\right)}}E\left(R^{\left(1\right)}\left(S_{N}^{\left(1\right)},A_{N}^{\left(1\right)}\right)\right)\\ = & \;\arg\max_{F_{N}^{\left(1\right)}}-\theta^{\left(1\right)}E{\left(S_{N}^{\left(1\right)}\right)}^{2}-\phi^{\left(1\right)}E{\left(F_{N}^{\left(1\right)}\left(S_{N}^{\left(1\right)}\right)\right)}^{2}. \end{array}$$

Since $p_{S_{N}^{\left(1\right)}}$ is given, the first term $-\theta^{\left(1\right)}E{\left(S_{N}^{\left(1\right)}\right)}^{2}$ is fixed. Note the upper bound on the second term $-\phi^{\left(1\right)}E{\left(F_{N}^{\left(1\right)}\left(S_{N}^{\left(1\right)}\right)\right)}^{2}\leq 0$. The upper bound can be achieved by the optimal deterministic privacy-preserving LQG policy:

(A2) $$\begin{array}{c} F_{N}^{(1)★}\left(s_{N}^{\left(1\right)}\right)=\kappa_{N}^{(1)★}s_{N}^{\left(1\right)}=0, \end{array}$$

where

(A3) $$\begin{array}{c} \kappa_{N}^{(1)★}=\frac{\frac{\lambda }{2\omega^{2}}\beta^{2}\kappa_{N}^{(0)*}-{\hat{\theta }}_{N+1}^{\left(1\right)}\alpha \beta }{\phi^{\left(1\right)}+{\hat{\theta }}_{N+1}^{\left(1\right)}\beta^{2}+\frac{\lambda }{2\omega^{2}}\beta^{2}}=0=\kappa_{N}^{(1)*}. \end{array}$$

Then, the maximum achievable objective of the final step is

(A4) $$\begin{array}{c} \max_{F_{N}^{\left(1\right)}}E\left(R^{\left(1\right)}\left(S_{N}^{\left(1\right)},A_{N}^{\left(1\right)}\right)\right)=-\theta^{\left(1\right)}E{\left(S_{N}^{\left(1\right)}\right)}^{2}=-{\hat{\theta }}_{N}^{\left(1\right)}E{\left(S_{N}^{\left(1\right)}\right)}^{2}. \end{array}$$

We then consider the sub-problem from the (N−1)-th step until the final step. Given a probability distribution $p_{S_{N-1}^{\left(1\right)}}$ and the optimal deterministic privacy-preserving LQG policy in the final step $F_{N}^{(1)★}$, the sub-optimization problem of the deterministic control policy $F_{N-1}^{\left(1\right)}$ is

(A5) $$\begin{array}{cc} F_{N-1}^{(1)★}= & \;\arg\max_{F_{N-1}^{\left(1\right)}}E\left(\sum_{i=N-1}^{N}R^{\left(1\right)}\left(S_{i}^{\left(1\right)},A_{i}^{\left(1\right)}\right)\right)-\lambda D\left(p_{S_{N}^{\left(1\right)}|S_{N-1}^{\left(1\right)}}||p_{S_{N}^{(0)*}|S_{N-1}^{(0)*}}\right)\\ = & \;\arg\max_{F_{N-1}^{\left(1\right)}}-\theta^{\left(1\right)}E{\left(S_{N-1}^{\left(1\right)}\right)}^{2}-\phi^{\left(1\right)}E{\left(A_{N-1}^{\left(1\right)}\right)}^{2}-{\hat{\theta }}_{N}^{\left(1\right)}E{\left(S_{N}^{\left(1\right)}\right)}^{2}\\ \; & \;\;\;-\lambda D\left(p_{S_{N}^{\left(1\right)}|S_{N-1}^{\left(1\right)}}||p_{S_{N}^{(0)*}|S_{N-1}^{(0)*}}\right)\\ = & \;\arg\max_{F_{N-1}^{\left(1\right)}}-\theta^{\left(1\right)}E{\left(S_{N-1}^{\left(1\right)}\right)}^{2}-\phi^{\left(1\right)}E{\left(F_{N-1}^{\left(1\right)}\left(S_{N-1}^{\left(1\right)}\right)\right)}^{2}-{\hat{\theta }}_{N}^{\left(1\right)}E{\left(S_{N}^{\left(1\right)}\right)}^{2}\\ \; & \;\;\;-\lambda E\left(\log\frac{\frac{1}{\sqrt{2\pi \omega^{2}}}\exp\left(-\frac{{\left(S_{N}^{\left(1\right)}-\alpha S_{N-1}^{\left(1\right)}-\beta F_{N-1}^{\left(1\right)}\left(S_{N-1}^{\left(1\right)}\right)\right)}^{2}}{2\omega^{2}}\right)}{\frac{1}{\sqrt{2\pi \omega^{2}}}\exp\left(-\frac{{\left(S_{N}^{\left(1\right)}-\alpha S_{N-1}^{\left(1\right)}-\beta \kappa_{N-1}^{(0)*}S_{N-1}^{\left(1\right)}\right)}^{2}}{2\omega^{2}}\right)}\right)\\ = & \;\arg\max_{F_{N-1}^{\left(1\right)}}-\theta^{\left(1\right)}E{\left(S_{N-1}^{\left(1\right)}\right)}^{2}-\phi^{\left(1\right)}E{\left(F_{N-1}^{\left(1\right)}\left(S_{N-1}^{\left(1\right)}\right)\right)}^{2}\\ \; & \;\;\;-{\hat{\theta }}_{N}^{\left(1\right)}E{\left(\alpha S_{N-1}^{\left(1\right)}+\beta F_{N-1}^{\left(1\right)}\left(S_{N-1}^{\left(1\right)}\right)+Z_{N-1}\right)}^{2}\\ \; & \;\;\;-\frac{\lambda }{2\omega^{2}}\beta^{2}E{\left(F_{N-1}^{\left(1\right)}\left(S_{N-1}^{\left(1\right)}\right)-\kappa_{N-1}^{(0)*}S_{N-1}^{\left(1\right)}\right)}^{2}\\ = & \;\arg\max_{F_{N-1}^{\left(1\right)}}-\left(\theta^{\left(1\right)}+{\hat{\theta }}_{N}^{\left(1\right)}\alpha^{2}+\frac{\lambda }{2\omega^{2}}\beta^{2}{\left(\kappa_{N-1}^{(0)*}\right)}^{2}\right)E{\left(S_{N-1}^{\left(1\right)}\right)}^{2}-{\hat{\theta }}_{N}^{\left(1\right)}E{\left(Z_{N-1}\right)}^{2}\\ \; & \;\;\;-\left(\phi^{\left(1\right)}+{\hat{\theta }}_{N}^{\left(1\right)}\beta^{2}+\frac{\lambda }{2\omega^{2}}\beta^{2}\right)E{\left(F_{N-1}^{\left(1\right)}\left(S_{N-1}^{\left(1\right)}\right)\right)}^{2}\\ \; & \;\;\;-\left(2{\hat{\theta }}_{N}^{\left(1\right)}\alpha \beta -\frac{\lambda }{\omega^{2}}\beta^{2}\kappa_{N-1}^{(0)*}\right)E\left(S_{N-1}^{\left(1\right)}F_{N-1}^{\left(1\right)}\left(S_{N-1}^{\left(1\right)}\right)\right)\\ = & \;\arg\max_{F_{N-1}^{\left(1\right)}}\int_{R}\left[-\left(\phi^{\left(1\right)}+{\hat{\theta }}_{N}^{\left(1\right)}\beta^{2}+\frac{\lambda }{2\omega^{2}}\beta^{2}\right){\left(F_{N-1}^{\left(1\right)}\left(s_{N-1}^{\left(1\right)}\right)\right)}^{2}\right.\\ \; & \;\;\;\left.-\left(2{\hat{\theta }}_{N}^{\left(1\right)}\alpha \beta -\frac{\lambda }{\omega^{2}}\beta^{2}\kappa_{N-1}^{(0)*}\right)s_{N-1}^{\left(1\right)}F_{N-1}^{\left(1\right)}\left(s_{N-1}^{\left(1\right)}\right)\right]p_{S_{N-1}^{\left(1\right)}}\left(s_{N-1}^{\left(1\right)}\right)ds_{N-1}^{\left(1\right)}\\ = & \;\int_{R}\arg\max_{F_{N-1}^{\left(1\right)}}\left[-\left(\phi^{\left(1\right)}+{\hat{\theta }}_{N}^{\left(1\right)}\beta^{2}+\frac{\lambda }{2\omega^{2}}\beta^{2}\right){\left(F_{N-1}^{\left(1\right)}\left(s_{N-1}^{\left(1\right)}\right)\right)}^{2}\right.\\ \; & \;\;\;\left.-\left(2{\hat{\theta }}_{N}^{\left(1\right)}\alpha \beta -\frac{\lambda }{\omega^{2}}\beta^{2}\kappa_{N-1}^{(0)*}\right)s_{N-1}^{\left(1\right)}F_{N-1}^{\left(1\right)}\left(s_{N-1}^{\left(1\right)}\right)\right]p_{S_{N-1}^{\left(1\right)}}\left(s_{N-1}^{\left(1\right)}\right)ds_{N-1}^{\left(1\right)}. \end{array}$$

Since ${\hat{\theta }}_{N}^{\left(1\right)}=\theta^{\left(1\right)}>0$, it follows that

(A6) $$\begin{array}{c} -\left(\phi^{\left(1\right)}+{\hat{\theta }}_{N}^{\left(1\right)}\beta^{2}+\frac{\lambda }{2\omega^{2}}\beta^{2}\right)<0. \end{array}$$

Given any $s_{N-1}^{\left(1\right)}\in R$, the objective of the inner optimization in (A5) is a concave quadratic function of $F_{N-1}^{\left(1\right)}\left(s_{N-1}^{\left(1\right)}\right)$. Therefore, we can obtain the optimal deterministic privacy-preserving LQG policy as

(A7) $$\begin{array}{c} F_{N-1}^{(1)★}\left(s_{N-1}^{\left(1\right)}\right)=\kappa_{N-1}^{(1)★}s_{N-1}^{\left(1\right)}, \end{array}$$

where

(A8) $$\begin{array}{c} \kappa_{N-1}^{(1)★}=\frac{\frac{\lambda }{2\omega^{2}}\beta^{2}\kappa_{N-1}^{(0)*}-{\hat{\theta }}_{N}^{\left(1\right)}\alpha \beta }{\phi^{\left(1\right)}+{\hat{\theta }}_{N}^{\left(1\right)}\beta^{2}+\frac{\lambda }{2\omega^{2}}\beta^{2}}. \end{array}$$

By using the optimal deterministic policies $F_{N-1:N}^{(1)★}$, the maximum achievable objective of the sub-problem is

(A9) $$\begin{array}{cc} \; & \max_{F_{N-1:N}^{\left(1\right)}}E\left(\sum_{i=N-1}^{N}R^{\left(1\right)}\left(S_{i}^{\left(1\right)},A_{i}^{\left(1\right)}\right)\right)-\lambda D\left(p_{S_{N}^{\left(1\right)}|S_{N-1}^{\left(1\right)}}||p_{S_{N}^{(0)*}|S_{N-1}^{(0)*}}\right)\\ \; & \;=-{\hat{\theta }}_{N-1}^{\left(1\right)}E{\left(S_{N-1}^{\left(1\right)}\right)}^{2}-{\hat{\theta }}_{N}^{\left(1\right)}E{\left(Z_{N-1}\right)}^{2}. \end{array}$$

The coefficient ${\hat{\theta }}_{N-1}^{\left(1\right)}$ can be specified as

(A10) $$\begin{array}{c} {\hat{\theta }}_{N-1}^{\left(1\right)}=\theta^{\left(1\right)}+\frac{\phi^{\left(1\right)}{\hat{\theta }}_{N}^{\left(1\right)}\alpha^{2}+\frac{\lambda }{2\omega^{2}}\beta^{2}{\left(\kappa_{N-1}^{(0)*}\right)}^{2}\phi^{\left(1\right)}+\frac{\lambda }{2\omega^{2}}\beta^{2}{\hat{\theta }}_{N}^{\left(1\right)}{\left(\alpha +\beta \kappa_{N-1}^{(0)*}\right)}^{2}}{\phi^{\left(1\right)}+{\hat{\theta }}_{N}^{\left(1\right)}\beta^{2}+\frac{\lambda }{2\omega^{2}}\beta^{2}}. \end{array}$$

It can be easily justified that ${\hat{\theta }}_{N-1}^{\left(1\right)}>0$ since ${\hat{\theta }}_{N}^{\left(1\right)}>0$. We now consider the sub-problem from the (N−2)-th step until the final step. Given a probability distribution $p_{S_{N-2}^{\left(1\right)}}$ and the optimal deterministic privacy-preserving LQG policies $F_{N-1:N}^{(1)★}$, the sub-optimization problem of the deterministic control policy $F_{N-2}^{\left(1\right)}$ is

(A11) $$\begin{array}{cc} F_{N-2}^{(1)★}= & \;\arg\max_{F_{N-2}^{\left(1\right)}}E\left(\sum_{i=N-2}^{N}R^{\left(1\right)}\left(S_{i}^{\left(1\right)},A_{i}^{\left(1\right)}\right)\right)-\lambda \sum_{i=N-1}^{N}D\left(p_{S_{i}^{\left(1\right)}|S_{i-1}^{\left(1\right)}}||p_{S_{i}^{(0)*}|S_{i-1}^{(0)*}}\right)\\ = & \;\arg\max_{F_{N-2}^{\left(1\right)}}-\theta^{\left(1\right)}E{\left(S_{N-2}^{\left(1\right)}\right)}^{2}-\phi^{\left(1\right)}E{\left(A_{N-2}^{\left(1\right)}\right)}^{2}-{\hat{\theta }}_{N-1}^{\left(1\right)}E{\left(S_{N-1}^{\left(1\right)}\right)}^{2}-{\hat{\theta }}_{N}^{\left(1\right)}E{\left(Z_{N-1}\right)}^{2}\\ \; & \;\;\;-\lambda D\left(p_{S_{N-1}^{\left(1\right)}|S_{N-2}^{\left(1\right)}}||p_{S_{N-1}^{(0)*}|S_{N-2}^{(0)*}}\right)\\ = & \;\arg\max_{F_{N-2}^{\left(1\right)}}-\theta^{\left(1\right)}E{\left(S_{N-2}^{\left(1\right)}\right)}^{2}-\phi^{\left(1\right)}E{\left(F_{N-2}^{\left(1\right)}\left(S_{N-2}^{\left(1\right)}\right)\right)}^{2}\\ \; & \;\;\;-{\hat{\theta }}_{N-1}^{\left(1\right)}E{\left(\alpha S_{N-2}^{\left(1\right)}+\beta F_{N-2}^{\left(1\right)}\left(S_{N-2}^{\left(1\right)}\right)+Z_{N-2}\right)}^{2}\\ \; & \;\;\;-\frac{\lambda }{2\omega^{2}}\beta^{2}E{\left(F_{N-2}^{\left(1\right)}\left(S_{N-2}^{\left(1\right)}\right)-\kappa_{N-2}^{(0)*}S_{N-2}^{\left(1\right)}\right)}^{2}-{\hat{\theta }}_{N}^{\left(1\right)}E{\left(Z_{N-1}\right)}^{2}\\ = & \;\int_{R}\arg\max_{F_{N-2}^{\left(1\right)}}\left[-\left(\phi^{\left(1\right)}+{\hat{\theta }}_{N-1}^{\left(1\right)}\beta^{2}+\frac{\lambda }{2\omega^{2}}\beta^{2}\right){\left(F_{N-2}^{\left(1\right)}\left(s_{N-2}^{\left(1\right)}\right)\right)}^{2}\right.\\ \; & \;\;\;\left.-\left(2{\hat{\theta }}_{N-1}^{\left(1\right)}\alpha \beta -\frac{\lambda }{\omega^{2}}\beta^{2}\kappa_{N-2}^{(0)*}\right)s_{N-2}^{\left(1\right)}F_{N-2}^{\left(1\right)}\left(s_{N-2}^{\left(1\right)}\right)\right]p_{S_{N-2}^{\left(1\right)}}\left(s_{N-2}^{\left(1\right)}\right)ds_{N-2}^{\left(1\right)}. \end{array}$$

Note that the objective functions in (A5) and (A11) have the same form. We have also proved that ${\hat{\theta }}_{N-1}^{\left(1\right)}>0$. Therefore, we can use the same arguments to obtain the optimal deterministic privacy-preserving LQG policy as

(A12) $$\begin{array}{c} F_{N-2}^{(1)★}\left(s_{N-2}^{\left(1\right)}\right)=\kappa_{N-2}^{(1)★}s_{N-2}^{\left(1\right)}, \end{array}$$

where

(A13) $$\begin{array}{c} \kappa_{N-2}^{(1)★}=\frac{\frac{\lambda }{2\omega^{2}}\beta^{2}\kappa_{N-2}^{(0)*}-{\hat{\theta }}_{N-1}^{\left(1\right)}\alpha \beta }{\phi^{\left(1\right)}+{\hat{\theta }}_{N-1}^{\left(1\right)}\beta^{2}+\frac{\lambda }{2\omega^{2}}\beta^{2}}, \end{array}$$

the maximum achievable objective of the sub-problem as

(A14) $$\begin{array}{cc} \; & \max_{F_{N-2:N}^{\left(1\right)}}E\left(\sum_{i=N-2}^{N}R^{\left(1\right)}\left(S_{i}^{\left(1\right)},A_{i}^{\left(1\right)}\right)\right)-\lambda \sum_{i=N-1}^{N}D\left(p_{S_{i}^{\left(1\right)}|S_{i-1}^{\left(1\right)}}||p_{S_{i}^{(0)*}|S_{i-1}^{(0)*}}\right)\\ \; & \;=-{\hat{\theta }}_{N-2}^{\left(1\right)}E{\left(S_{N-2}^{\left(1\right)}\right)}^{2}-{\hat{\theta }}_{N-1}^{\left(1\right)}E{\left(Z_{N-2}\right)}^{2}-{\hat{\theta }}_{N}^{\left(1\right)}E{\left(Z_{N-1}\right)}^{2}, \end{array}$$

and ${\hat{\theta }}_{N-2}^{\left(1\right)}>0$. We can further prove the optimal deterministic privacy-preserving LQG policies in the remaining steps and the maximum achievable weighted design objective of Agent B in Theorem 1 using the same arguments. □

## Appendix B

**Proof** **of Theorem 2.** The proof is based on the optimal deterministic privacy-preserving LQG policy of Agent B in Theorem 1. For all $1\leq i\leq \left\lceil \frac{N}{2}\right\rceil$ and x≥0, let

(A15) $$\begin{array}{c} J\left(x\right)=\lim_{N\to \infty }J_{N+1-i}\left(x\right)=\theta^{\left(1\right)}+\alpha^{2}x+\frac{\lambda }{2\omega^{2}}\beta^{2}{\left(\kappa^{(0)*}\right)}^{2}-\frac{{\left(\frac{\lambda }{2\omega^{2}}\beta^{2}\kappa^{(0)*}-\alpha \beta x\right)}^{2}}{\phi^{\left(1\right)}+\beta^{2}x+\frac{\lambda }{2\omega^{2}}\beta^{2}}, \end{array}$$

where the second equality follows from

(A16) $$\begin{array}{cc} \lim_{N\to \infty }\kappa_{i}^{(0)*}= & \;\lim_{N\to \infty }-\frac{{\tilde{\theta }}_{i+1}^{\left(0\right)}\alpha \beta }{\phi^{\left(0\right)}+{\tilde{\theta }}_{i+1}^{\left(0\right)}\beta^{2}}\\ = & \;-\frac{\alpha \beta \lim_{N\to \infty }\underset{N-i\;iterations}{\underset{︸}{L^{\left(0\right)}(L^{\left(0\right)}(\cdots (L^{\left(0\right)}(L^{\left(0\right)}}}\left({\tilde{\theta }}_{N+1}^{\left(0\right)}\right)))\cdots ))}{\phi^{\left(0\right)}+\beta^{2}\lim_{N\to \infty }\underset{N-i\;iterations}{\underset{︸}{L^{\left(0\right)}(L^{\left(0\right)}(\cdots (L^{\left(0\right)}(L^{\left(0\right)}}}\left({\tilde{\theta }}_{N+1}^{\left(0\right)}\right)))\cdots ))}\\ = & \;-\frac{{\tilde{\theta }}^{\left(0\right)}\alpha \beta }{\phi^{\left(0\right)}+{\tilde{\theta }}^{\left(0\right)}\beta^{2}}\\ = & \;\kappa^{(0)*}. \end{array}$$

When the model parameters satisfy the condition in (22), J(x) is a contraction mapping, i.e., there exists 0<γ<1 such that $\left|J\left(x\right)-J\left(x^{\prime }\right)\right|\leq \gamma \left|x-x^{\prime }\right|$ for all x≥0 and $x^{\prime }\geq 0$. From the Banach’s fixed point theorem, there is a unique fixed point ${\hat{\theta }}^{\left(1\right)}$ with respect to the contraction mapping *J* such that

(A17) $$\begin{array}{cc} {\hat{\theta }}^{\left(1\right)}= & \;\lim_{N\to \infty }J_{N}\left(J_{N-1}\left(\cdots \left(J_{2}\left(J_{1}\left({\hat{\theta }}_{N+1}^{\left(1\right)}\right)\right)\right)\cdots \right)\right)\\ = & \;\lim_{N\to \infty }J_{N}\left(J_{N-1}\left(\cdots \left(J_{N+2-\left\lceil \frac{N}{2}\right\rceil }\left(J_{N+1-\left\lceil \frac{N}{2}\right\rceil }\left({\hat{\theta }}_{\left\lceil \frac{N}{2}\right\rceil +1}^{\left(1\right)}\right)\right)\right)\cdots \right)\right)\\ = & \;\lim_{N\to \infty }\underset{\left\lceil \frac{N}{2}\right\rceil \;iterations}{\underset{︸}{J(J(\cdots (J(J}}\left({\hat{\theta }}_{\left\lceil \frac{N}{2}\right\rceil +1}^{\left(H\right)}\right)))\cdots ))\\ = & \;\theta^{\left(1\right)}+\alpha^{2}{\hat{\theta }}^{\left(1\right)}+\frac{\lambda }{2\omega^{2}}\beta^{2}{\left(\kappa^{(0)*}\right)}^{2}-\frac{{\left(\frac{\lambda }{2\omega^{2}}\beta^{2}\kappa^{(0)*}-\alpha \beta {\hat{\theta }}^{\left(1\right)}\right)}^{2}}{\phi^{\left(1\right)}+\beta^{2}{\hat{\theta }}^{\left(1\right)}+\frac{\lambda }{2\omega^{2}}\beta^{2}}. \end{array}$$

From (19)–(21), (A16), and (A17), it is easy to verify the time-invariant optimal deterministic privacy-preserving LQG policy of Agent B in (24)–(25) and the asymptotic weighted design objective rate in (26). □

## Appendix C

**Proof** **of Theorem 3.** The proof is similar as that of Theorem 1. We first consider the sub-problem of the final step. Given a probability distribution $p_{S_{N}^{\left(1\right)}}$, the final step optimization problem of the linear Gaussian random policy $F_{N}^{\left(1\right)}$ with parameters $\kappa_{N}^{\left(1\right)}$ and $\delta_{N}^{2}$ is

(A18) $$\begin{array}{cc} \left(\kappa_{N}^{(1)★},\delta_{N}^{2★}\right)= & \;\arg\max_{\kappa_{N}^{\left(1\right)}\in R,\delta_{N}^{2}\in R_{\geq 0}}E\left(R^{\left(1\right)}\left(S_{N}^{\left(1\right)},A_{N}^{\left(1\right)}\right)\right)\\ = & \;\arg\max_{\kappa_{N}^{\left(1\right)}\in R,\delta_{N}^{2}\in R_{\geq 0}}-\theta^{\left(1\right)}E{\left(S_{N}^{\left(1\right)}\right)}^{2}-\phi^{\left(1\right)}E{\left(A_{N}^{\left(1\right)}\right)}^{2}\\ = & \;\arg\max_{\kappa_{N}^{\left(1\right)}\in R,\delta_{N}^{2}\in R_{\geq 0}}\left(-\theta^{\left(1\right)}-\phi^{\left(1\right)}{\left(\kappa_{N}^{\left(1\right)}\right)}^{2}\right)E{\left(S_{N}^{\left(1\right)}\right)}^{2}-\phi^{\left(1\right)}\delta_{N}^{2}. \end{array}$$

It is obvious the optimal parameters are $\kappa_{N}^{(1)★}=0$ and $\delta_{N}^{2★}=0$, i.e., the optimal linear Gaussian random policy reduces to the optimal deterministic privacy-preserving LQG policy in the final step. Similarly, we then consider the sub-problem from the (N−1)-th step until the final step. Given a probability distribution $p_{S_{N-1}^{\left(1\right)}}$ and the optimal linear Gaussian random policy in the final step $F_{N}^{(1)★}$, the sub-optimization problem of the linear Gaussian random policy $F_{N-1}^{\left(1\right)}$ with parameters $\kappa_{N-1}^{\left(1\right)}$ and $\delta_{N-1}^{2}$ is

(A19) $$\begin{array}{cc} \; & \left(\kappa_{N-1}^{(1)★},\delta_{N-1}^{2★}\right)\\ \; & =\;\arg\max_{\kappa_{N-1}^{\left(1\right)}\in R,\delta_{N-1}^{2}\in R_{\geq 0}}E\left(\sum_{i=N-1}^{N}R^{\left(1\right)}\left(S_{i}^{\left(1\right)},A_{i}^{\left(1\right)}\right)\right)-\lambda D\left(p_{S_{N}^{\left(1\right)}|S_{N-1}^{\left(1\right)}}||p_{S_{N}^{(0)*}|S_{N-1}^{(0)*}}\right)\\ \; & =\;\arg\max_{\kappa_{N-1}^{\left(1\right)}\in R,\delta_{N-1}^{2}\in R_{\geq 0}}-\theta^{\left(1\right)}E{\left(S_{N-1}^{\left(1\right)}\right)}^{2}-\phi^{\left(1\right)}E{\left(A_{N-1}^{\left(1\right)}\right)}^{2}-{\hat{\theta }}_{N}^{\left(1\right)}E{\left(S_{N}^{\left(1\right)}\right)}^{2}\\ \; & \;\;\;-\lambda D\left(p_{S_{N}^{\left(1\right)}|S_{N-1}^{\left(1\right)}}||p_{S_{N}^{(0)*}|S_{N-1}^{(0)*}}\right)\\ \; & =\;\arg\max_{\kappa_{N-1}^{\left(1\right)}\in R,\delta_{N-1}^{2}\in R_{\geq 0}}-\theta^{\left(1\right)}E{\left(S_{N-1}^{\left(1\right)}\right)}^{2}-\phi^{\left(1\right)}E{\left(A_{N-1}^{\left(1\right)}\right)}^{2}-{\hat{\theta }}_{N}^{\left(1\right)}E{\left(S_{N}^{\left(1\right)}\right)}^{2}\\ \; & \;\;\;-\lambda E\left(\log\frac{\frac{1}{\sqrt{2\pi \left(\omega^{2}+\beta^{2}\delta_{N-1}^{2}\right)}}\exp\left(-\frac{{\left(S_{N}^{\left(1\right)}-\alpha S_{N-1}^{\left(1\right)}-\beta \kappa_{N-1}^{\left(1\right)}S_{N-1}^{\left(1\right)}\right)}^{2}}{2\left(\omega^{2}+\beta^{2}\delta_{N-1}^{2}\right)}\right)}{\frac{1}{\sqrt{2\pi \omega^{2}}}\exp\left(-\frac{{\left(S_{N}^{\left(1\right)}-\alpha S_{N-1}^{\left(1\right)}-\beta \kappa_{N-1}^{(0)*}S_{N-1}^{\left(1\right)}\right)}^{2}}{2\omega^{2}}\right)}\right)\\ \; & =\;\arg\max_{\kappa_{N-1}^{\left(1\right)}\in R,\delta_{N-1}^{2}\in R_{\geq 0}}-\theta^{\left(1\right)}E{\left(S_{N-1}^{\left(1\right)}\right)}^{2}-\phi^{\left(1\right)}E{\left(\kappa_{N-1}^{\left(1\right)}S_{N-1}^{\left(1\right)}+W_{N-1}^{\left(1\right)}\right)}^{2}\\ \; & \;\;\;-{\hat{\theta }}_{N}^{\left(1\right)}E{\left(\alpha S_{N-1}^{\left(1\right)}+\beta \kappa_{N-1}^{\left(1\right)}S_{N-1}^{\left(1\right)}+\beta W_{N-1}^{\left(1\right)}+Z_{N-1}\right)}^{2}\\ \; & \;\;\;-\frac{\lambda }{2\omega^{2}}\beta^{2}{\left(\kappa_{N-1}^{\left(1\right)}-\kappa_{N-1}^{(0)*}\right)}^{2}E{\left(S_{N-1}^{\left(1\right)}\right)}^{2}-\frac{\lambda }{2\omega^{2}}\beta^{2}\delta_{N-1}^{2}\\ \; & \;\;\;-\frac{\lambda }{2}\log\frac{\omega^{2}}{\omega^{2}+\beta^{2}\delta_{N-1}^{2}}\\ \; & =\;\arg\max_{\kappa_{N-1}^{\left(1\right)}\in R,\delta_{N-1}^{2}\in R_{\geq 0}}-\left(\phi^{\left(1\right)}+{\hat{\theta }}_{N}^{\left(1\right)}\beta^{2}+\frac{\lambda }{2\omega^{2}}\beta^{2}\right)E{\left(S_{N-1}^{\left(1\right)}\right)}^{2}{\left(\kappa_{N-1}^{\left(1\right)}\right)}^{2}\\ \; & \;\;\;-\left(2{\hat{\theta }}_{N}^{\left(1\right)}\alpha \beta -\frac{\lambda }{\omega^{2}}\beta^{2}\kappa_{N-1}^{(0)*}\right)E{\left(S_{N-1}^{\left(1\right)}\right)}^{2}\kappa_{N-1}^{\left(1\right)}\\ \; & \;\;\;-\left(\theta^{\left(1\right)}+{\hat{\theta }}_{N}^{\left(1\right)}\alpha^{2}+\frac{\lambda }{2\omega^{2}}\beta^{2}{\left(\kappa_{N-1}^{(0)*}\right)}^{2}\right)E{\left(S_{N-1}^{\left(1\right)}\right)}^{2}-{\hat{\theta }}_{N}^{\left(1\right)}\omega^{2}\\ \; & \;\;\;-\left(\phi^{\left(1\right)}+{\hat{\theta }}_{N}^{\left(1\right)}\beta^{2}+\frac{\lambda }{2\omega^{2}}\beta^{2}\right)\delta_{N-1}^{2}-\frac{\lambda }{2}\log\frac{\omega^{2}}{\omega^{2}+\beta^{2}\delta_{N-1}^{2}}. \end{array}$$

(A19) consists of two independent optimizations: the optimization of $\kappa_{N-1}^{\left(1\right)}\in R$ and the optimization of $\delta_{N-1}^{2}\in R_{\geq 0}$. Since ${\hat{\theta }}_{N}^{\left(1\right)}>0$, it follows that

(A20) $$\begin{array}{c} -\left(\phi^{\left(1\right)}+{\hat{\theta }}_{N}^{\left(1\right)}\beta^{2}+\frac{\lambda }{2\omega^{2}}\beta^{2}\right)E{\left(S_{N-1}^{\left(1\right)}\right)}^{2}<0. \end{array}$$

The optimization of $\kappa_{N-1}^{\left(1\right)}\in R$ has a concave quadratic objective. Then we can obtain the optimal linear coefficient as

(A21) $$\begin{array}{c} \kappa_{N-1}^{(1)★}=\frac{\frac{\lambda }{2\omega^{2}}\beta^{2}\kappa_{N-1}^{(0)*}-{\hat{\theta }}_{N}^{\left(1\right)}\alpha \beta }{\phi^{\left(1\right)}+{\hat{\theta }}_{N}^{\left(1\right)}\beta^{2}+\frac{\lambda }{2\omega^{2}}\beta^{2}}. \end{array}$$

The optimization of $\delta_{N-1}^{2}\in R_{\geq 0}$ has a decreasing objective. Then, the optimal variance is $\delta_{N-1}^{2★}=0$. Therefore, the optimal linear Gaussian random policy reduces to the optimal deterministic privacy-preserving LQG policy in the (N−1)-th step. We then consider the sub-problem from the (N−2)-th step until the final step. Given a probability distribution $p_{S_{N-2}^{\left(1\right)}}$ and the optimal linear Gaussian random policies $F_{N-1:N}^{(1)★}$, the sub-optimization problem of the linear Gaussian random policy $F_{N-2}^{\left(1\right)}$ with parameters $\kappa_{N-2}^{\left(1\right)}$ and $\delta_{N-2}^{2}$ is

(A22) $$\begin{array}{cc} \; & \left(\kappa_{N-2}^{(1)★},\delta_{N-2}^{2★}\right)\\ \; & =\;\arg\max_{\kappa_{N-2}^{\left(1\right)}\in R,\delta_{N-2}^{2}\in R_{\geq 0}}E\left(\sum_{i=N-2}^{N}R^{\left(1\right)}\left(S_{i}^{\left(1\right)},A_{i}^{\left(1\right)}\right)\right)-\lambda \sum_{i=N-1}^{N}D\left(p_{S_{i}^{\left(1\right)}|S_{i-1}^{\left(1\right)}}||p_{S_{i}^{(0)*}|S_{i-1}^{(0)*}}\right)\\ \; & =\;\arg\max_{\kappa_{N-2}^{\left(1\right)}\in R,\delta_{N-2}^{2}\in R_{\geq 0}}-\theta^{\left(1\right)}E{\left(S_{N-2}^{\left(1\right)}\right)}^{2}-\phi^{\left(1\right)}E{\left(A_{N-2}^{\left(1\right)}\right)}^{2}-{\hat{\theta }}_{N-1}^{\left(1\right)}E{\left(S_{N-1}^{\left(1\right)}\right)}^{2}-{\hat{\theta }}_{N}^{\left(1\right)}\omega^{2}\\ \; & \;\;\;-\lambda D\left(p_{S_{N-1}^{\left(1\right)}|S_{N-2}^{\left(1\right)}}||p_{S_{N-1}^{(0)*}|S_{N-2}^{(0)*}}\right)\\ \; & =\;\arg\max_{\kappa_{N-2}^{\left(1\right)}\in R,\delta_{N-2}^{2}\in R_{\geq 0}}-\theta^{\left(1\right)}E{\left(S_{N-2}^{\left(1\right)}\right)}^{2}-\phi^{\left(1\right)}E{\left(\kappa_{N-2}^{\left(1\right)}S_{N-2}^{\left(1\right)}+W_{N-2}^{\left(1\right)}\right)}^{2}\\ \; & \;\;\;-{\hat{\theta }}_{N-1}^{\left(1\right)}E{\left(\alpha S_{N-2}^{\left(1\right)}+\beta \kappa_{N-2}^{\left(1\right)}S_{N-2}^{\left(1\right)}+\beta W_{N-2}^{\left(1\right)}+Z_{N-2}\right)}^{2}-{\hat{\theta }}_{N}^{\left(1\right)}\omega^{2}\\ \; & \;\;\;-\frac{\lambda }{2\omega^{2}}\beta^{2}{\left(\kappa_{N-2}^{\left(1\right)}-\kappa_{N-2}^{(0)*}\right)}^{2}E{\left(S_{N-2}^{\left(1\right)}\right)}^{2}-\frac{\lambda }{2\omega^{2}}\beta^{2}\delta_{N-2}^{2}\\ \; & \;\;\;-\frac{\lambda }{2}\log\frac{\omega^{2}}{\omega^{2}+\beta^{2}\delta_{N-2}^{2}}\\ \; & =\;\arg\max_{\kappa_{N-2}^{\left(1\right)}\in R,\delta_{N-2}^{2}\in R_{\geq 0}}-\left(\phi^{\left(1\right)}+{\hat{\theta }}_{N-1}^{\left(1\right)}\beta^{2}+\frac{\lambda }{2\omega^{2}}\beta^{2}\right)E{\left(S_{N-2}^{\left(1\right)}\right)}^{2}{\left(\kappa_{N-2}^{\left(1\right)}\right)}^{2}\\ \; & \;\;\;-\left(2{\hat{\theta }}_{N-1}^{\left(1\right)}\alpha \beta -\frac{\lambda }{\omega^{2}}\beta^{2}\kappa_{N-2}^{(0)*}\right)E{\left(S_{N-2}^{\left(1\right)}\right)}^{2}\kappa_{N-2}^{\left(1\right)}\\ \; & \;\;\;-\left(\theta^{\left(1\right)}+{\hat{\theta }}_{N-1}^{\left(1\right)}\alpha^{2}+\frac{\lambda }{2\omega^{2}}\beta^{2}{\left(\kappa_{N-2}^{(0)*}\right)}^{2}\right)E{\left(S_{N-2}^{\left(1\right)}\right)}^{2}-{\hat{\theta }}_{N-1}^{\left(1\right)}\omega^{2}-{\hat{\theta }}_{N}^{\left(1\right)}\omega^{2}\\ \; & \;\;\;-\left(\phi^{\left(1\right)}+{\hat{\theta }}_{N-1}^{\left(1\right)}\beta^{2}+\frac{\lambda }{2\omega^{2}}\beta^{2}\right)\delta_{N-2}^{2}-\frac{\lambda }{2}\log\frac{\omega^{2}}{\omega^{2}+\beta^{2}\delta_{N-2}^{2}}. \end{array}$$

Note that the objective functions in (A19) and (A22) have the same form. Therefore, we can use the same arguments to show that the optimal linear Gaussian random policy reduces to the optimal deterministic privacy-preserving LQG policy in the (N−2)-th step, i.e., $\delta_{N-2}^{2★}=0$ and

(A23) $$\begin{array}{c} \kappa_{N-2}^{(1)★}=\frac{\frac{\lambda }{2\omega^{2}}\beta^{2}\kappa_{N-2}^{(0)*}-{\hat{\theta }}_{N-1}^{\left(1\right)}\alpha \beta }{\phi^{\left(1\right)}+{\hat{\theta }}_{N-1}^{\left(1\right)}\beta^{2}+\frac{\lambda }{2\omega^{2}}\beta^{2}}. \end{array}$$

We can further prove the optimal linear Gaussian random policies in the remaining steps reduce to the optimal deterministic privacy-preserving LQG policies based on the same arguments. □
